# Cell wall peptidoglycan in *Mycobacterium tuberculosis*: An Achilles’ heel for the TB-causing pathogen

**DOI:** 10.1093/femsre/fuz016

**Published:** 2019-06-10

**Authors:** Arundhati Maitra, Tulika Munshi, Jess Healy, Liam T Martin, Waldemar Vollmer, Nicholas H Keep, Sanjib Bhakta

**Affiliations:** Mycobacteria Research Laboratory, Institute of Structural and Molecular Biology, Department of Biological Sciences, Birkbeck, University of London, Malet Street, London WC1E 7HX, UK; Mycobacteria Research Laboratory, Institute of Structural and Molecular Biology, Department of Biological Sciences, Birkbeck, University of London, Malet Street, London WC1E 7HX, UK; Department of Pharmaceutical and Biological Chemistry, UCL School of Pharmacy, 29–39 Brunswick Square, London WC1N 1AX, UK; Mycobacteria Research Laboratory, Institute of Structural and Molecular Biology, Department of Biological Sciences, Birkbeck, University of London, Malet Street, London WC1E 7HX, UK; The Centre for Bacterial Cell Biology, Institute for Cell and Molecular Biosciences, Newcastle University, Richardson Road, Newcastle upon Tyne, NE2 4AX, UK; Mycobacteria Research Laboratory, Institute of Structural and Molecular Biology, Department of Biological Sciences, Birkbeck, University of London, Malet Street, London WC1E 7HX, UK; Mycobacteria Research Laboratory, Institute of Structural and Molecular Biology, Department of Biological Sciences, Birkbeck, University of London, Malet Street, London WC1E 7HX, UK

**Keywords:** Tuberculosis (TB), cell envelope, peptidoglycan, metabolism, drug-target validation, antibiotic resistance, antibacterial

## Abstract

Tuberculosis (TB), caused by the intracellular pathogen *Mycobacterium tuberculosis*, remains one of the leading causes of mortality across the world. There is an urgent requirement to build a robust arsenal of effective antimicrobials, targeting novel molecular mechanisms to overcome the challenges posed by the increase of antibiotic resistance in TB. *Mycobacterium tuberculosis* has a unique cell envelope structure and composition, containing a peptidoglycan layer that is essential for maintaining cellular integrity and for virulence. The enzymes involved in the biosynthesis, degradation, remodelling and recycling of peptidoglycan have resurfaced as attractive targets for anti-infective drug discovery. Here, we review the importance of peptidoglycan, including the structure, function and regulation of key enzymes involved in its metabolism. We also discuss known inhibitors of ATP-dependent Mur ligases, and discuss the potential for the development of pan-enzyme inhibitors targeting multiple Mur ligases.

## Teaser

A comprehensive, investigative review that highlights key enzymes involved in mycobacterial cell wall peptidoglycan metabolism as novel anti-infective drug targets.

## INTRODUCTION

Tuberculosis (TB) is a leading cause of mortality in the world today and is caused by the bacterial pathogen *Mycobacterium tuberculosis*. Despite innovations in diagnostics and improved access to health care, the global burden of TB remains substantial with around 10 million new cases of infection and 1.6 million deaths reported due to TB in 2017 alone (WHO 2018). An estimated 457 000 cases reported in 2017 presently harbour multi-drug resistant TB (MDR-TB), 8.5% of which are expected to be extensively drug resistant TB (XDR-TB) (WHO 2018), reflecting the urgent need to design and develop novel drugs to treat TB.

The success of *M. tuberculosis* as a pathogen and its innate resistance to many antimicrobial drugs can be attributed in part to its unique cell wall structure, which has low permeability for many drugs and possesses a large number of efflux pumps (Jarlier and Nikaido 1994, Brennan and Nikaido 1995). The cell wall is a defining characteristic of all bacteria. Amongst the many purposes it serves, maintaining the cell-shape and withstanding turgor are key. The varying properties of the bacterial cell wall, especially the thickness of the peptidoglycan (PG), impart different stain-retention properties to the bacterial cell and enable us to categorise most bacteria into Gram-positive, Gram-negative and acid-fast. The presence of PG across nearly all bacteria indicates that it was likely to have been present in their last common ancestor (Errington 2013). Importantly, PG is essential for bacterial cell survival in most environments, thus making it a good target for anti-infective therapy.

**Figure 1.**
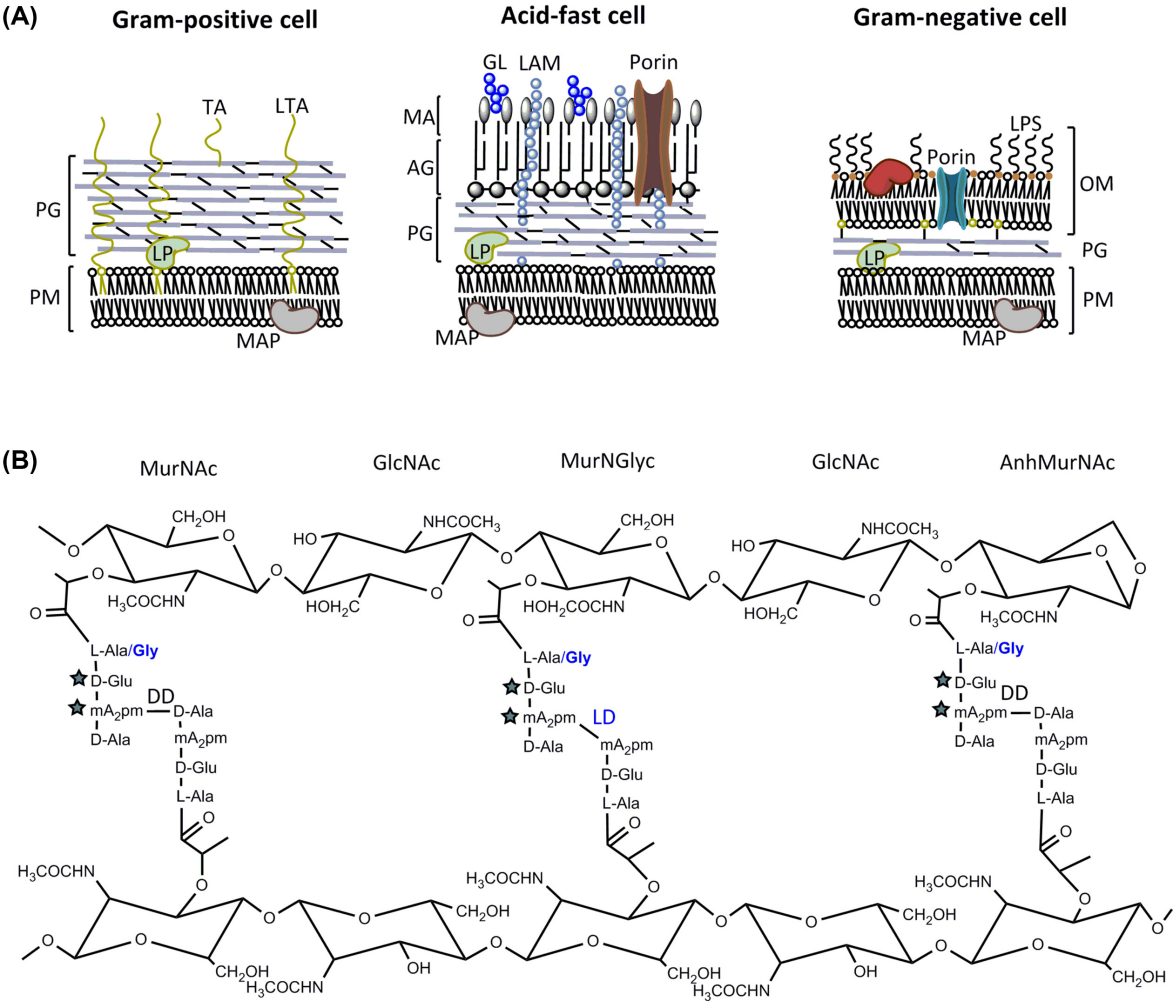
**(A)**, Differences in the cell envelope architecture of Gram-positive, acid-fast and Gram-negative bacteria. AG, arabinogalactan; GL, glycolipid; LAM, lipoarabinomannan; LP, lipoprotein; LPS, lipopolysaccharide; LTA, lipoteichoic acid; MA, mycolic acid; MAP, membrane-associated protein; OM, outer membrane; PG, peptidoglycan; PM, plasma membrane; TA, teichoic acid . **(B)**, PG structure revealing the unique features present in mycobacterial cells highlighted in blue. The stars depict residues that undergo amidation.

Mycobacteria belong to the diverse family of Actinobacteria. The main components of the mycobacterial cell wall are the PG layer, mycolic acid (MA) and arabinogalactan (AG). The mycobacterial cell wall resembles both the Gram-positive and Gram-negative cell envelope by having a PG layer nearly as thick as the former and an outer, waxy layer mimicking the outer membrane of the latter (Fig.   1A). The cell wall of mycobacteria plays a key role in intrinsic antibiotic resistance and virulence (Forrellad *et al*. 2013; Becker and Sander 2016) but it is unclear why and how its complex structure evolved. Vincent *et al*. recently proposed that the ‘mycomembrane’ evolved by successive horizontal acquisition of genes; explaining the narrow distribution of the AG and MA biosynthetic genes in some lineages of the Actinobacteria that evolved later, while the PG genes are broadly distributed (Vincent *et al*. 2018).

PG is a dynamic structure but the factors that regulate its composition and biogenesis in the slow-growing intra-cellular pathogen *M. tuberculosis* are not well understood. The mycobacterial PG plays a key role in the cell's growth, cell–cell communication and in the initiation of the host immune response. The cell envelope of some model bacteria such as *Escherichia coli*, have long been the focus of extensive research; however, these organisms do not serve as appropriate models when studying the unique physiology and biochemistry of *M. tuberculosis*. Here, we review the different stages of PG metabolism in *M. tuberculosis*, its importance for survival of the bacilli and the potential therapeutic targets it offers for the design of new anti-infective drugs.

### Modifications of cell wall PG in *M. tuberculosis*

Lying outside the cytoplasmic membrane of *M. tuberculosis*, the PG layer is covalently attached to AG which itself serves as an attachment site for unique MAs (Fig. 1A). This is referred to as the cell wall core, or as the MA-AG-PG complex (MAPc) (Brennan 2003). Beyond and interspersed within the MAPc are free lipids, phosphatidyl inositol mannosides (PIM), lipomannans (LM) and lipoarabinomannans (LAM). As observed in other microorganisms (Jankute *et al*. 2015), the disruption of PG synthesis or digestion of the PG layer leads to the formation of L-form bacilli that display a variety of cell shapes and morphology in mycobacteria (Udou, Ogawa and Mizuguchi 1982).

PG is made of glycan strands of alternating, β(1→4)-linked *N*-acetylglucosamine (Glc*N*Ac) and *N*-acetylmuramic acid (Mur*N*Ac) residues. Adjacent glycan strands are connected by short peptide stems with the sequence l-alanyl-γ-d-isoglutamyl-*meso*-diaminopimelate-d-alanyl-d-alanine and linked to the d-lactoyl residue of each Mur*N*Ac. Peptides protruding from adjacent glycan strands can be cross-linked between the carboxyl group of d-Ala at the fourth position and the ε-amino group of the *meso*-diaminopimelate (*m*DAP) in the third position.

While the basic structure of PG is conserved, it is important to note that the PG in mycobacteria undergoes several modifications (Fig. 1B). PG isolated from *M. tuberculosis* and *M. smegmatis* contains both *N*-acetylmuramic acid (Mur*N*Ac) and *N*-glycolylmuramic acid (Mur*N*Glyc), whereas most other bacteria, including *M. leprae*, exclusively contain Mur*N*Ac (Mahapatra *et al*. 2005a, Petit *et al*. 1969; Mahapatra *et al*. 2008). The presence of Mur*N*Glyc increases the resistance of mycobacterial PG to lysozyme (Raymond *et al*. 2005). Modifications such as *N*-deacetylation or *O*-acetylation of GlcNAc and MurNAc respectively, as observed in other pathogenic bacteria (Vollmer 2008) have not been reported in mycobacteria. Consequently, the deletion of the hydroxylase responsible for *N*-glycolylation, *namH*, increases the sensitivity towards β-lactams and lysozyme in *M. smegmatis* (Raymond *et al*. 2005). Glycolylated PG fragments are more potent at inducing NOD2-mediated host responses such as production of tumour necrosis factor α (Hansen *et al*. 2014), which may appear to be counter-productive for the success of an intracellular pathogen. However, the enhanced stimulation of the host might enable *M. tuberculosis* to recruit phagocytic cells, its ecological niche within the host and facilitate transmission to a new host.

The peptide stems in PG undergo modifications such as amidation of the α-carboxylic group of d-isoglutamate (d-iGlu) and the ε-carboxylic group of the mDAP residues (Kotani *et al*. 1970; Mahapatra *et al*. 2008). d-iGlu amidation is required for the functioning of PG transpeptidases that cross-link the peptides to form a robust PG (Figueiredo *et al*. 2012; Zapun *et al*. 2013) whereas mDAP amidation is involved in modulating PG hydrolysis thereby maintaining the integrity of the cell wall (Nakel, Ghuysen and Kandler 1971; Dajkovic *et al*. 2017). These modifications reduce the net negative charge of the cell wall, which impairs the efficacy of antimicrobial factors such as lysozyme, antimicrobial peptides and cell wall acting antibiotics (Girardin *et al*. 2003b; Roychowdhury, Wolfert and Boons 2005). In the PG of *M. leprae*, Gly replaces l-Ala in position 1 of the peptide stem, and an additional Gly residue is attached to *m*DAP (Mahapatra *et al*. 2008).

The PG of *M. tuberculosis* contains a particularly high percentage (70%–80%) of cross-linked peptides (Wietzerbin *et al*. 1974), compared to 40%–50% in *E. coli* (Glauner, Holtje and Schwarz 1988). One-third of the peptide cross-links are DD-(or 4→3) bonds between d-Ala and *m*DAP, and about two-thirds are LD-(or 3→3) bonds linking two *m*DAP residues (Wietzerbin *et al*. 1974; Hett and Rubin 2008) (Fig. 1B). Although LD-crosslinks are relatively rare in many other bacterial species, recent reports have estimated them to account for 80% of the peptide cross-links in the PG of stationary phase or dormant cells of *M. tuberculosis* (Lavollay *et al*. 2008). The LD-cross-links are also enhanced to 15%–20% of all cross-links in the PG of stationary phase cells of *E. coli*, but are significantly reduced (5% of all cross-links) in exponentially growing cells (Pisabarro, de Pedro and Vazquez 1985; Glauner, Holtje and Schwarz 1988; Templin, Ursinus and Holtje 1999).

Interestingly, the pattern of PG incorporation into the cell wall of *M. tuberculosis* is different compared to the PG of other rod-shaped bacteria (Daniel and Errington 2003; Hett and Rubin 2008). Most rod-shaped bacilli such as *E. coli* and *Bacillus subtilis* elongate by inserting nascent PG along the lateral sides of the cell (den Blaauwen *et al*. 2008). An actin-like protein, MreB, positions the PG-elongation machinery along the short axis of the cell so that glycan strands are inserted circumferentially thus maintaining the rod-shape (Domínguez-Escobar *et al*. 2011). In contrast, mycobacteria lack MreB and other proteins important for lateral wall elongation and instead incorporate nascent PG at both poles of the cell (Thanky, Young and Robertson 2007; Kieser and Rubin 2014). Mycobacteria exhibit differential incorporation of PG at the two cell poles (old versus new cell pole) at certain stages of the cell cycle (before/after cytokinesis); this allows for asymmetric cell division and results in a heterogeneous population of cells (Kieser and Rubin 2014; Botella *et al*.2017b). Baranowski *et al*. reported that the ability of the mycobacterial cell to generate LD-cross-links (discussed later in this review) is essential in maintaining the rod-shape (Baranowski *et al*. 2018). However, whether this serves as the sole mechanism regulating cell shape is yet to be confirmed.

### Imaging PG

The recent application of fluorescence and atomic force microscopy techniques together with new probes for labelling have significantly advanced our understanding of the dynamics of PG synthesis (Radkov *et al*. 2018). Localisation studies using GFP-labelled PG biosynthetic proteins (Joyce *et al*. 2012; Grzegorzewicz *et al*. 2016), fluorescent d-amino acids (Siegrist *et al*. 2013; Meniche *et al*. 2014; Boutte *et al*. 2016; Schubert *et al*. 2017; Botella *et al*. 2017b; Rodriguez‐Rivera *et al*. 2018) and cell wall targeting antibiotics (Thanky, Young and Robertson 2007; Kastrinsky and Barry 2010) have further emphasized the importance of PG remodelling in cell elongation, septum formation and cell division. Aldridge *et al*. used microfluidics-based live-cell imaging technique to show that the heterogeneous daughter cell populations differ in their susceptibility to antibiotics (Aldridge *et al*. 2012). Botella *et al*. compared the spatiotemporal dynamics of PG synthesis in *M. smegmatis* and *M. tuberculosis* using super-resolution microscopy combined with fluorescent d-alanine analogues (FDAAs) (Botella *et al*. 2017b). They reported that FDAAs are predominantly incorporated at one of the two poles in *M. smegmatis*, whereas *M. tuberculosis* shows variation in polar dominance depending on the stage in cell cycle. FDAAs are also incorporated along the lateral wall upon damage due to muramidase activity (Garcia-Heredia *et al*. 2018). Furthermore, fluorescent intracellular membrane domain (IMD)-associated protein reporters revealed that mycobacteria can spatiotemporally coordinate its membrane domain in response to metabolic requirements under different growth conditions (Hayashi *et al*. 2018). These recent reports on membrane compartmentalisation of PG synthesis and its metabolism during the cell cycle in *M. tuberculosis* will help to understand mechanisms allowing bacteria to escape host defence systems and to characterise drug targeting steps that have remained elusive before.

### Biosynthesis and maturation of PG

The nucleotide precursors of PG were first isolated from *Staphylococcus aureus* in 1952 (Park 1952). Since then the various steps involved in the biosynthesis of PG have been extensively studied in a number of species. The PG biosynthetic pathway in *M. tuberculosis*, however, remains largely uncharacterised. In this work we follow the generally accepted assumption that mycobacteria synthesise PG precursors in a similar way to the model bacterium *E. coli* using enzymes that are homologous to the ones found in the latter. However, as a slow-growing intra-cellular pathogen with varied physiological states, mycobacteria have PG enzymes that differ with respect to structure and regulation compared to their counterparts in *E. coli*. This review attempts to highlight these critical features. Details of the enzymes discussed throughout the review have been listed in Table S1 (Supporting Information).

### PG biosynthesis: the cytoplasmic steps

**Figure 2.**
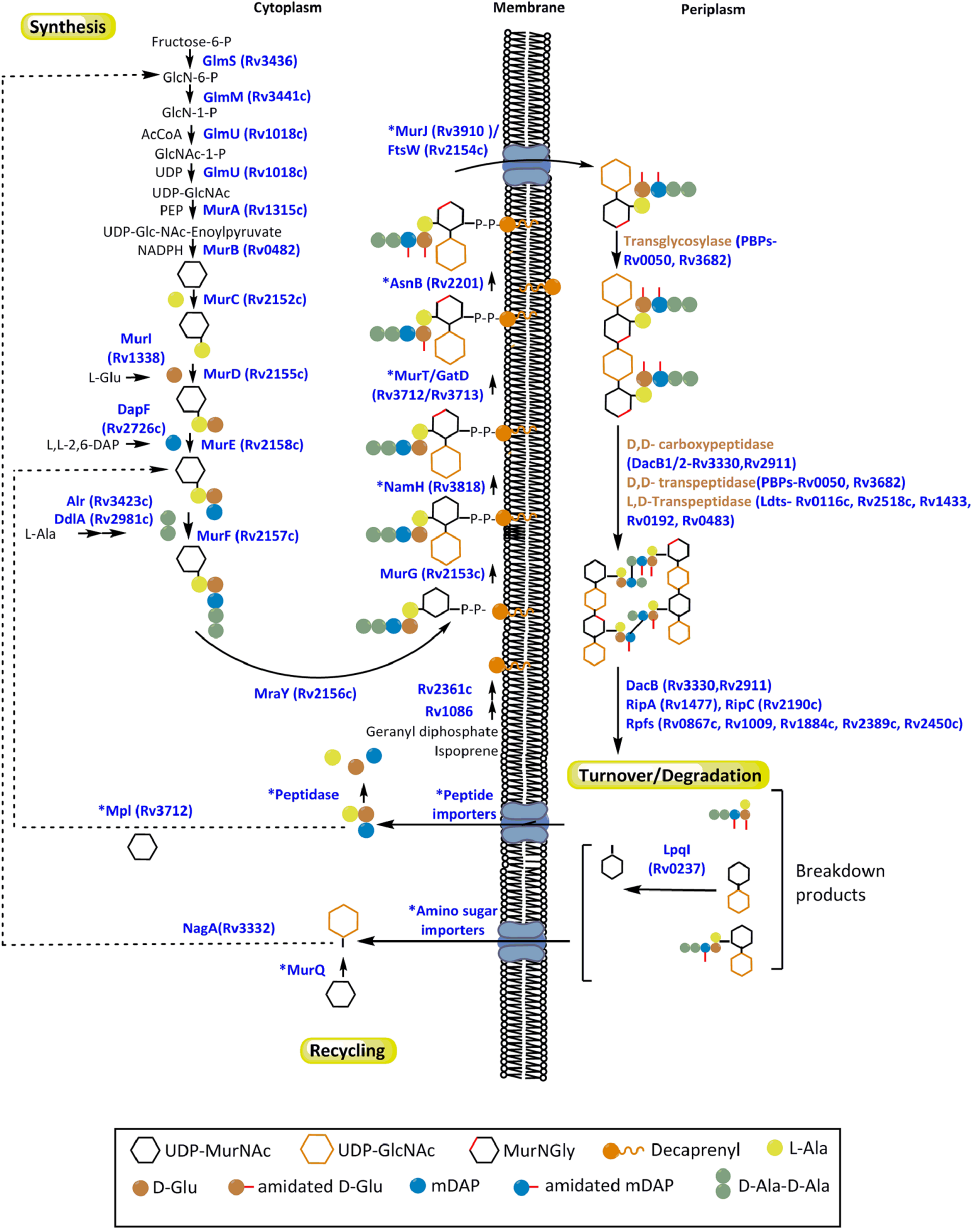
An overview of PG biosynthesis, degradation and recycling pathways representing information currently known in *Mycobacterium* species. While there has been plethora of information on the PG metabolism in other bacteria, identification of mycobacterial proteins involved in this process has been limited so far. ‘*’ marks enzymes that have not been experimentally established. This includes proteins that are putative or completely unknown and uncertainty on when the enzymatic step occurs in the pathway. The redundancy in the PG hydrolases makes it difficult to assemble a comprehensive list within a figure and only the selected enzymes discussed in this article are highlighted. Recycling of PG in mycobacteria is an especially underexplored area compared to other bacteria and may offer interesting insight into homeostasis mechanisms within the cell.

PG biosynthesis begins with the conversion of fructose-6-phosphate to UDP-*N*-acetylglucosamine (UDP-Glc*N*Ac), which is catalysed by the enzymes GlmS, GlmM and GlmU (van Heijenoort 2001a) (Fig. 2). Of these three enzymes, only GlmU has been characterised in *M. tuberculosis* (Zhang *et al*. 2009; Jagtap *et al*. 2012). GlmU is a bifunctional acetyltransferase/uridyltransferase, which converts glucosamine-1-phosphate (Glc*N*-1-P) to *N*-acetylglucosamine-1-phosphate (Glc*N*Ac-1-P) and subsequently catalyses the formation of UDP-Glc*N*Ac from Glc*N*Ac-1-P and uridine triphosphate (Zhang *et al*. 2009; Jagtap *et al*. 2012; Li *et al*. 2012). The reaction mechanism of the acetyl transfer by GlmU provides useful insights for drug design, such as the conformational changes of the protein structure upon interaction with the ligand (Craggs *et al*. 2018). *Mycobacterium tuberculosis* GlmU is trimeric in solution, whereby each monomer folds into two distinct domains. The N-terminal domain has a typical uridyltransferase fold based on a dinucleotide-binding Rossmann fold and is similar to that observed in the reported structure for GlmU from *Streptococcus pneumoniae* (Sulzenbacher *et al*. 2001). The C-terminal domain has a left-handed parallel β-helix fold forming the acetyltransferase active site containing contributions from all three subunits as is characteristic of other acetyltransferase enzymes. However, the GlmU from *M. tuberculosis* is 6–8 fold less active than that of GlmU from *E. coli*. Additionally, as *M. tuberculosis* GlmU lacks free cysteines in the acetyl-CoA binding site or any solvent-accessible cysteines elsewhere, it retains its acetyltransferase activity even in the absence of reducing agents and in the presence of a thiol-reactive reagent; both of which render the *E. coli* enzyme inactive (Pompeo, van Heijenoort and Mengin-Lecreulx 1998; Zhang *et al*. 2009). The crystal structure of *M. tuberculosis* GlmU reveals a unique 30-residue extension which forms a short helix at the C-terminus and is involved in substrate binding (Jagtap *et al*. 2012). The C-terminus of the enzyme is also involved in auto-regulation through phosphorylation by the serine/threonine protein kinase PknB (Parikh *et al*. 2009). Dziadek *et al*. recently reported that GlmU interacts with host immune factor IL-8, thereby increasing mycobacterial attachment to neutrophils and facilitating infection (Dziadek *et al*. 2016). How a cytosolic bacterial protein serves as a receptor binding entity still requires clarification, however, the enzyme has garnered interest for drug development. The enzyme is essential for *M. tuberculosis* and it depletion results in severe growth defects *in vitro* and reduced bacillary loads in mice models (Soni *et al*. 2015). Further experiments showed that the uridyl- and acetyl-transferase activities are individually essential for the pathogen thus inhibitors designed for either active site would be effective. Also, as the synthesis of UDP-Glc*N*Ac occurs via a different enzymatic route in eukaryotes compared to prokaryotes, any treatment designed against the latter would be expected to have little effect on the former.

MurA (UDP-*N*-acetylglucosamine enolpyruvyl transferase) and MurB (UDP-*N*-acetylenolpyruvyl-glucosamine reductase) catalyse the conversion of UDP-*N*-acetylglucosamine (UDP-Glc*N*Ac) to UDP-*N*-acetylmuramate (UDP-Mur*N*Ac). MurA catalyses the transfer of the enolpyruvyl moiety of phosphoenolpyruvate (PEP) to UDP-Glc*N*Ac (De Smet *et al*. 1999; Xu *et al*. 2014). MurB (Benson *et al*. 1993) is an NADPH dependent oxidoreductase that reduces the enolpyruvate moiety to d-lactate resulting in the formation of UDP-Mur*N*Ac. While mycobacteria and Gram-negative bacteria like *E. coli* have a single copy of the *murA* gene (Brown *et al*. 1995), a second transferase gene, *murA2* occurs as a duplicate copy in low G + C Gram-positive organisms such as *S. pneumoniae* (Du *et al*. 2000). Interestingly, both copies perform the same function and can substitute for one another. The purpose for such redundancy at the first committed step of PG biosynthesis may be to accommodate differential regulation (Du *et al*. 2000). UDP-Mur*N*Ac binds to *E. coli* MurA with high affinity, suggesting an inhibitory or regulatory role in PG biosynthesis (Mizyed *et al*. 2005). CwlM, an essential, cytoplasmic protein and hypothetical PG amidase, is another regulator of MurA (Boutte *et al*. 2016). Phophorylated CwlM increases the activity of MurA nearly 30 fold. CwlM is phosphorylated only in the presence of surplus nutrients, thereby revealing a possible mechanism by which the pathogen correlates PG synthesis with the availability of nutrients. Reducing the PG synthesis rate also has implications for drug susceptibility, therefore mechanisms which can activate CwlM and MurA may help to target the ‘persister’ population of *M. tuberculosis* and reduce treatment duration, one of the main causes of patient non-compliance. The regulatory role of CwlM has since been investigated further and its influences at later stages of PG synthesis have also been revealed and will be discussed in the following section.

Homology modelling, molecular dynamics and molecular docking studies on the mycobacterial MurA and MurB enzymes revealed active site residues and helped identifying potential inhibitors (Babajan *et al*. 2011; Kumar *et al*. 2011). The crystal structure of *M. tuberculosis* MurB with FAD as the prosthetic group was recently reported (Eniyan *et al*. 2018). MurB is a type I UDP-Glc*N*AcEP reductase and shares conserved domains with MurB from *E. coli* and *Pseudomonas aeruginosa*. The nicotinamide and the enol pyruvyl moieties aligned well in *M. tuberculosis* MurB upon superimposition with the other MurB structures and show domain III to adopt a more open conformation on binding to the substrates. There are known inhibitors of MurB from Gram-positive and Gram-negative bacteria, however, whether these are active against *M. tuberculosis* MurB has not been investigated (Bronson *et al*. ; Yang *et al*. ).

**Figure 3.**
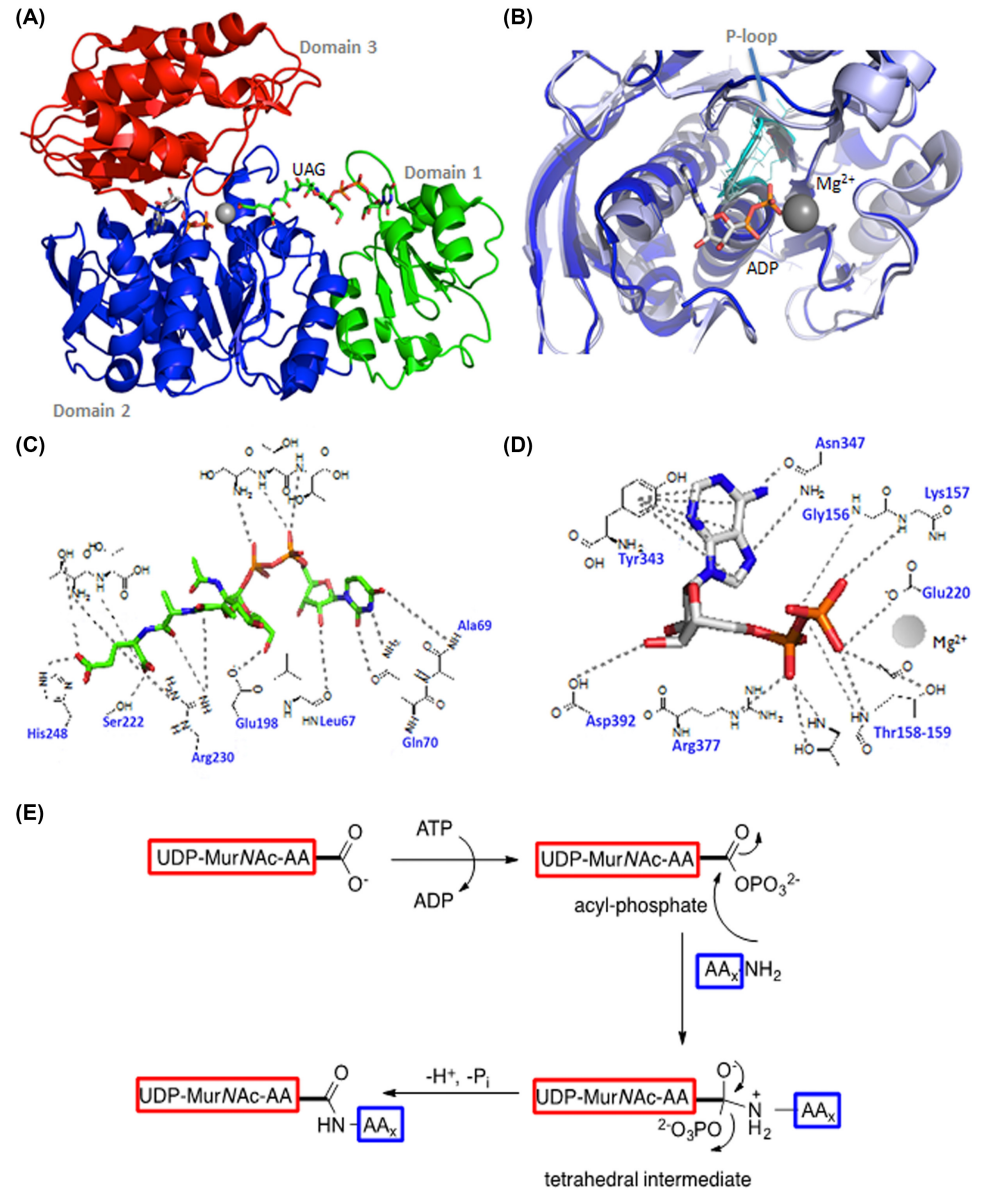
**(A)**, Cartoon representation of the crystal structure of MurE from *M. tuberculosis*. The UDP substrate binding domain 1 is shown in green, the central ATPase domain 2 is shown in blue and the amino acid substrate binding domain 3 is shown in red. Image generated using PyMOL (PDB code: 2XJA). **(B)**, Overlay of domain 2 from MurE from *E. coli* (PDB code: 1E8C, light blue) and *M. tuberculosis* (PDB code: 2XJA, dark blue). The P-loops are highlighted in cyan. **(C)**, Interaction map of the UDP-MurNAc substrate with MurE from *M. tuberculosis*. **(D)**, Interaction map of ADP with MurE from *M. tuberculosis*. **(E)**, The reaction mechanism of Mur ligases. The ‘AA’ in the UDP-Mur*N*Ac-AA substrate represents the peptide stems of varying lengths whereas the AA_x_.NH_2_ represents the incoming amino acid.

The assembly of the peptide stem of the PG monomer unit (UDP-Mur*N*Glyc or UDP-Mur*N*Ac-pentapeptide) is catalysed by a series of four essential and structurally related ATP-dependent Mur ligases MurC, MurD, MurE and MurF which, in *M. tuberculosis*, catalyse the addition of l-Ala, d-Glu, *m*DAP and d-Ala- d-Ala, respectively, with the concomitant hydrolysis of ATP to ADP and inorganic phosphate (Fig.   2) (Basavannacharya *et al*. 2010a; Basavannacharya *et al*. 2010b; Munshi *et al*. 2013; Eniyan *et al*. 2016). The mycobacterial Mur ligases catalyse amide bond formation *via* a similar reaction mechanism to that seen in the *E. coli* enzymes, i.e. the formation of an activated acylphosphate derivative of UDP-Mur*N*Ac followed by a nucleophilic attack by the amino group of the amino acid or dipeptide, ultimately resulting in the formation of a peptide bond and release of inorganic phosphate (Anderson *et al*. 1996; Falk *et al*. 1996; Liger *et al*. 1996; Tanner *et al*. 1996; Vaganay *et al*. 1996; Bertrand *et al*. 1999) (Fig. 3E).

All ATP dependent Mur ligases (Mur synthetases) contain common structural motifs despite their low amino acid sequence identities. ATP-dependent Mur ligases can be described as containing three distinct domains: (i) an N-terminal Rossmann-type α/β fold domain that binds to the UDP-substrate, (ii) a central ATPase domain (with a highly conserved WalkerA-like motif, TG[T/S]XGK[T/S(2)]) and (iii) a C-terminal domain that binds the appropriate amino acid (Smith 2006) (Fig. 3).

The first amino acid of the peptide stem (l-Ala) is ligated onto UDP-Mur*N*Ac by MurC. In *M. leprae*, l-Ala is replaced by Gly. Interestingly, *M. tuberculosis* MurC is able to incorporate both l-Ser and Gly into the growing peptide chain *in vitro* (Munshi *et al*. 2013), although it is questionable whether the UDP substrates containing these amino acids would be accepted by MurD in the subsequent reaction. This substrate flexibility has also been observed in some other organisms. Addition of d-Glu by MurD is followed by incorporation of *m*DAP onto the growing peptide chain by MurE. While over-expression of *M. tuberculosis* MurE in *E. coli* and *Pseudomonas putida* does not affect growth, a lytic effect was observed when the *S. aureus* MurE (which incorporates l-Lys instead of *m*DAP) was overexpressed in *E. coli* (Mengin-Lecreulx *et al*. 1999). In all *m*DAP-specific MurE ligases, an arginine residue at position 416 binds to the free end of *m*DAP, whereas this residue is replaced by an alanine or asparagine in all l-Lys-specific MurE ligases (Dementin 2001). MurE from *M. tuberculosis* contains three domains, similar to that reported for *E. coli*. However, unlike its *E. coli* homologue, the mycobacterial MurE coordinates a magnesium ion with the UDP-MurNAc-l-Ala-d-Glu substrate (Basavannacharya *et al*. 2010a; Basavannacharya *et al*. 2010b). The last two amino acids of the stem peptide are added by MurF as the dipeptide d-Ala-d-Ala Interestingly, MurF from *E. coli* accepts muropeptides with either *m*DAP or l-Lys with similar efficiencies and its *N*-terminus is distinct from the other Mur ligases (Yan *et al*. 2000).

The entire MurA-F pathway is unique to prokaryotes and all corresponding genes are single-copy and essential in *M. tuberculosis* (Sassetti, Boyd and Rubin 2003; Griffin *et al*. 2011; DeJesus *et al*. 2017) making them attractive therapeutic targets. MurC-F share conserved residues and mode of action, and are thus amenable to multi-target therapy. Development of high-throughput activity assays as well as a one-pot assay that screens for inhibitors of more than one enzyme in the Mur pathway could aid drug discovery (Basavannacharya *et al*. 2010a; Eniyan *et al*. 2016).

In addition to ATP-dependent Mur ligases, the enzymes catalysing the formation of substrates for Mur ligase reactions are also key components of PG biosynthetic machinery. MurI is a glutamate racemase, which provides the d-Glu required for PG synthesis. Unlike in *Bacillus subtilis* there is only one gene coding for MurI in *M. tuberculosis*. The available crystal structure of MurI (Poen *et al*. 2016) and new inhibitors of the enzyme (Prosser *et al*. 2016) strengthen its potential as druggable target in the pathogen. However, several issues question the value of MurI as a drug target. Homogeneous MurI purifies as a stable dimer that is enzymatically inactive *in vitro*. It is presumed that activator molecules such as UDP-Mur*N*Ac-l-Ala may dissociate the dimer to generate the active enzyme as observed in the case of *E. coli* MurI (Ho *et al*. 1995). This inability to extract and purify an enzymatically active form of the enzyme from heterologous expression systems poses a significant bottleneck for the development of high-throughput inhibitor screening assays. Additionally, there is some ambiguity over its essentiality (Sassetti, Boyd and Rubin 2003; Harth *et al*. 2005; Griffin *et al*. 2011; Morayya *et al*. 2015). Mortuza *et al*. identified a d‐amino acid transaminase in *M. smegmatis* that could complement the activity of MurI allowing *murI* mutants to grow in the absence of d-Glu (Mortuza *et al*. 2018). Whether its homologue in *M. tuberculosis* also retains d‐amino acid transaminase activity is not known. The presence of enzymes with overlapping functions indicates that resistance would develop easily against glutamate racemase inhibitors. However, as TB chemotherapy is always a combination, the rise of resistance may be controlled by administering a combination of drugs that work synergistically, such as glutamate racemase inhibitors and d-cycloserine (David 2001).

mDAP is synthesised from aspartate in eight biosynthetic steps (Usha *et al*. 2012). The enzymes in the pathway are essential and thus have the potential to become drug targets. Alpha-ketopimelic acid inhibits the activity of DapA (dihydrodipicolinate synthase) in the micromolar range indicating that it is a druggable target (Shrivastava *et al*. 2016). *Mycobacterium tuberculosis* DapE was found to be insensitive to the known DapE inhibitor L-captopril (Usha *et al*. 2016). Finally, DapF in *Chlamydia trachomatis* is bifunctional and involved in both mDAP synthesis and the racemisation of glutamate (Liechti *et al*. 2018). Whether the DapF from *M. tuberculosis* could also function as a d-Glu racemase is unknown. This essential enzyme converts ll-DAP into *m*DAP. Although little variation was observed in the two-domain α/β-fold of the apo structure of DapF from *M. tuberculosis*, when compared with DapF from *Haemophilus* and *Bacillus* species, it was noted that the *M. tuberculosis* DapF backbone is stabilised *via* a tyrosine residue specific to mycobacterial DAP epimerases. Any change of the interactions this residue is involved in would destabilize DapF (Usha *et al*. 2009) thereby providing a rationale for design of anti-tubercular specific DapF inhibitors.

The di-peptide substrate for MurF (d-Ala-d-Ala) is synthesised by the alanine racemase Alr, followed by the d-Ala-d-Ala ligase Ddl. Alr is a pyridoxal-5-phosphate-containing enzyme (van Heijenoort 2001b) and its crystal structure shows a homodimer with the domains adopting a similar fold to that observed in Alr structures from other species (LeMagueres *et al*. 2005). However, the observed hinge angle between the domains is different for Alr from *M. tuberculosis*. The interest in Alr as a drug target has been dampened by conflicting reports on its essentiality in *M. smegmatis* and *M. tuberculosis*. While Milligan *et al*. found deletion mutants require a steady supply ofd-Ala for growth, insertion mutants that did not show growth defects in the absence of d-Ala were selected for by Marshall *et al*. (Milligan *et al*. 2007; Awasthy *et al*. 2012, Marshall *et al*. 2017). Therefore, it is hypothesised that the function of Alr can be substituted by an unidentified transaminase that converts pyruvate and d-Glu to d-Ala so Alr-specific inhibitors would likely have no anti-mycobacterial effect.

Ddl catalyses the ATP-dependent ligation reaction to generate the dipeptide d-Ala-d-Ala. While only one copy of *ddl* was identified in mycobacteria, two *ddl* genes are present in *E. coli* (Zawadzke, Bugg and Walsh 1991). Ddl exists as a dimer, in which each monomer contains three domains with the ligand binding site formed at the intersection of these three domains (Bruning *et al*. 2011). The apo form of Ddl from *M. tuberculosis* is unique in that in the absence of substrate it adopts a closed conformation that has to undergo a significant structural rearrangement to bind to the substrates. The closed conformation of Ddl offers many unique binding pockets adjacent to the active site that may be useful for drug design. Ligand binding and catalytic activity, therefore, requires significantly greater structural rearrangement compared to orthologues from other species such as *S. aureus*. Biochemical analyses and classical enzyme kinetic studies of Ddl from *M. tuberculosis* revealed the mechanism of inhibition by d-cycloserine—knowledge that could be useful in the design of future drugs (Prosser and de Carvalho 2013). Currently, d-cycloserine is known to target Ddl and Alr in *M. tuberculosis*, with strong inhibition observed in the case of Ddl and weaker inhibition seen with Alr.

### PG biosynthesis: the membrane-associated steps

A lipid carrier is essential for the transport of the hydrophilic PG precursors across the hydrophobic cell membrane. Mycobacteria differ from *E. coli* in terms of the molecule that serves as the lipid carrier (decaprenyl phosphate versus undecaprenyl-phosphate) and in the synthesis of this molecule. In fact, even within mycobacteria there are marked differences within this pathway. Based on sequence homology it is presumed that *M. smegmatis* genome codes for 4 to 10 prenyl diphosphate synthases, whereas *M. tuberculosis* could contain up to 4 synthases (Crick *et al*. 2000). Rv1086 and Rv2361 are two distinct prenyl diphosphate synthases, a ω,E,Z-farnesyl diphosphate synthase (Z-FPPS) and a decaprenyl diphosphate (DecaPP) synthase, respectively (Schulbach, Brennan and Crick 2000). These act sequentially to synthesise decaprenyl diphosphate from isopentenyl diphosphate and E-geranyl diphosphate (Schulbach, Brennan and Crick 2000; Schulbach *et al*. 2001; Kaur, Brennan and Crick 2004). Rv1086 adds one isoprene unit to geranyl diphosphate, generating the 15-carbon product (E,Z-farnesyl diphosphate). Rv2361c then processively adds a further seven isoprene units to E,Z-farnesyl diphosphate to generate the 50-carbon prenyl diphosphate. The crystal structures of the enzymes show that they are closely related, which partly explains how Rv2361 can compensate for the deletion of Rv1086 (Schulbach *et al*. 2001; Wang *et al*. 2008). The decaprenyl diphosphate, synthesised *de novo* at the cytoplasmic side of the cell membrane or released by the polymerisation of PG on the periplasmic face of the membrane, must be dephosphorylated for the transfer of the activated sugar molecule from lipid I. The dephosphorylation of decaprenyl diphosphate to decaprenyl phosphate is performed by membrane-bound pyrophosphatases. In spite of multiple undecaprenyl pyrophosphate phosphatases (Upp) identified in *E. coli* (BacA, PgpB, YbjG and LpxT) the majority of this activity (75%) is attributed to BacA (UppP); which also confers bacitracin resistance in *E. coli* (El Ghachi *et al*. 2005; Bickford and Nick 2013). PgpB and BacA interact with PBP1B (which polymerises PG) and stimulate its activity by dephosphorylation of the released undecaprenyl pyrophosphate (Hernández-Rocamora *et al*. 2018). The crystal structure of *E. coli* BacA shows a unique topology raising the possibility of its involvement as a lipid carrier flippase in addition to being a phosphatase that could potentially accept substrates from the cytoplasm as well as the periplasm (Workman, Worrall and Strynadka 2018). Despite the redundancy of the phosphatases, deletion mutants lacking *bacA* show marked phenotypic effects such as attenuation of virulence in *Staphylococcus aureus* and *Streptococcus pneumoniae* in mice models, and impaired biofilm formation in *M. smegmatis* (Chalker *et al*. 2000; Röse, Kaufmann and Daugelat 2004). However, the *M. tuberculosis* homologue of BacA was found to have no role in either acid resistance or in virulence in mice models (Darby *et al*. 2011). This suggests that there may be other enzymes in *M. tuberculosis* with decaprenyl pyrophosphate phosphatase activity. The synthesis and recycling of the lipid carrier is essential for the membrane-bound steps of PG synthesis as discussed below and is a verified target for antibiotics such as bacitracin (Siewert and Strominger 1967).

The first membrane step in the biosynthesis of PG involves the phospho-*N*-acetylmuramoyl-pentapeptide transferase or translocase MraY. MraY catalyses the transfer of the phospho-Mur*N*Ac-pentapeptide moiety of UDP-Mur*N*Ac-pentapeptide to the acceptor decaprenyl phosphate, yielding Mur*N*Ac-(pentapeptide)-pyrophosphorylundecaprenol or lipid I. This proceeds *via* a covalent intermediate, which is formed upon nucleophilic attack by an aspartate residue on the β-phosphate of the UDP-Mur*N*Ac-pentapeptide resulting in the release of UMP (Chung *et al*. 2013). While *E. coli* uses the undecaprenyl lipid, *M. tuberculosis* solely uses the decaprenyl lipid, however, both decaprenyl and heptaprenyl acceptors have been identified for *M. smegmatis* (Takayama, Schnoes and Semmler 1973). Comprehensive biochemical or structural studies on mycobacterial MraY have not been reported. However, active site mapping of MraY from *E. coli* and comparison with MraY from other Gram-negative and Gram-positive bacteria revealed five conserved hydrophilic sequences located on the cytoplasmic side of the membrane containing invariant amino acids (Al-Dabbagh *et al*. 2008). An X-ray crystal structure for MraY from *Aquifex aeolicus* and a method to quantify MraY activity has provided further insight into the active site residues (Chung *et al*. 2013). Improvements of the biochemical assay to quantify MraY activity (Siricilla *et al*. 2014) is a significant asset in the effort to develop antimycobacterial agents targeting this protein.

Following the synthesis of lipid I, the *N*-acetylglucosamine transferase MurG catalyses the transfer of GlcNAc from UDP-Glc*N*Ac to lipid I, yielding Glc*N*Ac-Mur*N*Ac-(pentapeptide)-pyrophosphoryl-decaprenol or lipid II.

MurT and GatD are responsible for the amidation of the d-Glu residue in the peptide stem of both, lipid I and lipid II in *Staphylococcus aureus* and *Streptococcus pneumoniae* (Figueiredo *et al*. 2012; Munch *et al*. 2012). The arrangement of the *murT* and *gatD* genes in an operon is conserved across various bacterial families (Figueiredo *et al*. 2012) and has also been confirmed in mycobacteria (Maitra *et al*., unpublished results). Crystal structures of the enzymes from *S. aureus* and *S. pneumoniae* reveal that the pair form a stable, functional heterodimer wherein GatD is responsible for the sequestration of the glutamine donor and its subsequent deamination and MurT is a Mur ligase-like protein that allows for the d-iGlu in the stem peptide to be amidated to d-iGln and utilises an ATP in the process (Leisico *et al*. 2018; Morlot *et al*. 2018; Noldeke *et al*. 2018). While GatD is inactive in the absence of MurT, the latter can functionally substitute the role of MurE (Munch *et al*. 2012; Leisico *et al*. 2018). Amidation of PG plays a crucial role in PG synthesis and maturation. It was hypothesised that amidation could facilitate the translocation of lipid II across the cytoplasmic membrane as a consequence of the reduction of polarity (Munch *et al*. 2012). It has also been reported that non-amidated lipid II is an inefficient substrate for transpeptidation reactions in *S. pneumoniae* (Zapun *et al*. 2013). Amidated muropeptides are recognized by PknB in *M. tuberculosis* and through the action of the kinase could regulate cell growth, division and PG turnover (Mir *et al*. 2011).

The homologous *murT*/*gatD* genes in *M. tuberculosis* are essential for *in vitro* growth of the pathogen (Sassetti, Boyd and Rubin 2003). The essentiality of MurT/GatD and the requirement for complex formation for activity may be exploited for designing drug scaffolds.

Mycobacterial lipid II also contains amidated mDAP residues (Mahapatra *et al*. 2005b). In *Corynebacterium glutamicum* and *B. subtilis*, *ltsA* and *asnB* encode for the amidotransferases responsible for the amidation of mDAP (Levefaudes *et al*. 2015; Dajkovic *et al*. 2017). LtsA and AsnB belong to the growing family of glutamine amidotransferases (such as GatD) whose members catalyse amide nitrogen transfer from glutamine to various specific acceptor substrates (mDAP in this case). Most of the investigations into the role of mDAP amidation have focussed on the host immune response modulation of such modification. Nucleotide-binding oligomerisation domain-containing protein 1 (NOD1) is a pattern recognition receptor that recognizes Mur*N*Ac-tripeptide fragments containing mDAP and activates the innate immune system (Girardin *et al*. 2003b). Amidation of mDAP enables evasion of recognition by NOD1 by interacting with residues away from the NOD1-leucine-rich repeat (LRR) recognition region (Roychowdhury, Wolfert and Boons 2005; Wolfert, Roychowdhury and Boons 2007; Vijayrajratnam *et al*. 2016; Wang *et al*. 2016). Unlike d-Glu amidation, the modification of mDAP residue does not influence the pattern of insertion of PG precursors into the cell wall or the overall cross-linking levels (Levefaudes *et al*. 2015; Dajkovic *et al*. 2017). However, the amidation of the mDAP residues in mycobacteria is essential for the formation of LD-cross-links (Ngadjeua *et al*. 2018). Additionally, the presence of zones of uncontrolled PG degradation in the *ΔasnB* mutant of *B. subtilis* suggests that mDAP amidation controls the activity of hydrolytic enzymes such as autolysins (Dajkovic *et al*. 2017). Once the modifications have been introduced, lipid II is flipped to the outer face of the cytoplasmic membrane.

The translocation of lipid II across the cytoplasmic membrane has been subject to some controversy, with the role of lipid II flippase having being contested between FtsW and MurJ. The evidence supporting FtsW as the lipid II flippase comes from an assay performed by Mohammadi *et al*. (Mohammadi *et al*. 2011) on an *in vitro* reconstitution system, in which it was observed that FtsW, and not MurJ, was able to translocate fluorescently labelled lipid II across the membrane of bacterial membrane vesicles. It was later suggested by the same research group that lipid II may be translocated via a pore-like structure in FtsW (Mohammadi *et al*. 2014). Conversely, it has been suggested based on genetic analyses and biochemical assays with RodA, that proteins from the SEDS family may be glycosyltranferases (GTases) rather than lipid II flippases (Meeske *et al*. 2016; Emami *et al*. 2017). However, FtsW was not active as GTase in another study in which it was also observed that FtsW, but not MurJ, binds lipid (Leclercq *et al*. 2017). However, FtsW does in fact have GTase activity, but only when in complex with its cognate penicillin-binding protein (Taguchi *et al*. 2019).

The initial hypothesis for MurJ as the lipid II flippase was based on its essentiality and the observation that PG synthesis stops and precursors accumulate upon depletion of the *murJ* gene in *E. coli* (Inoue *et al*. 2008; Ruiz 2008). A colicin M-based cellular assay suggested that MurJ might flip lipid II across the membrane (Sham *et al*. 2014). Native mass spectroscopy showed that MurJ binds lipid II and that this binding is affected by cardiolipin (Bolla *et al*. 2018). The crystal structures of MurJ from *Thermosipho afticanus* (Kuk, Mashalidis and Lee 2017) and *E. coli* (Zheng *et al*. 2018) confirmed similarity to MOP family of transporters and further rationalised possible mechanisms by which MurJ is likely to transport lipid II across the membrane. However, until there is direct evidence with the mycobacterial protein, the identity of the flippase in the pathogen remains uncertain.

Both PG precursor synthesis and their transport are regulated by PknB-mediated phosphorylation of CwlM (introduced earlier as regulating MurA activity). CwlM exists in two forms within the mycobacterial cell. The non-phosphorylated CwlM is membrane-associated, interacts with MurJ and presumably enables it to transport lipid II (Turapov *et al*. 2018). The phosphorylated CwlM is cytoplasmic and interacts with FhaA and MurA. The authors hypothesise that on binding to unpolymerised PG precursors in the periplasmic region, PknB undergoes autophosphorylation and in turn phophorylates its substrate CwlM, which dissociates from MurJ thereby reducing the flipping of PG precursors. Direct phosphorylation of MurJ by PknB was also proposed to produce the same outcome.

A number of compound classes bind to extracytoplasmic lipid II and prevent PG polymerisation. Glycopeptides such as vancomycin (MIC_Mtb_- 65 µg/mL) bind to d-Ala-d-Ala in lipid II and nascent PG. Antimicrobial peptides such as nisin (MIC_Mtb_- > 60 µg/mL), teixobactin (MIC_Mtb_- 0.125 µg/mL) and malacidin (MIC_Mtb_- not determined) bind primarily to the pyrophosphate moiety of lipid II and inhibit PG synthesis.

### PG biosynthesis: linkage in mature PG

Once the precursors are translocated to the periplasmic space, lipid II is used as the substrate for the subsequent polymerisation reactions and the formation of mature PG. The final stages of PG maturation occurs in the periplasm, carried out by transglycosylases and transpeptidases, which are often bifunctional penicilloyl serine transferases or penicillin binding proteins (PBPs) (Crick, Mahapatra and Brennan 2001; Sauvage *et al*. 2008), resulting in the formation mature of PG chains and peptide with DD-cross-links (Goffin and Ghuysen 1998). The class A and B PBPs synthesize PG; whereas the class C PBPs are PG hydrolases with either DD-endopeptidase or DD-carboxypeptidase activity. Both reactions provide the tetrapeptide substrates for LD-transpeptidases. Class A PBPs (in mycobacteria PBP1 and PBP1A, encoded by *ponA1 and ponA2*, respectively) consist of bifunctional enzymes with an N-terminal glycosyltransferase and a C-terminal DD-transpeptidase (penicillin-binding) domain (Mahapatra *et al*. 2000; Bhakta and Basu 2002; Patru and Pavelka 2010). Class B PBPs (in mycobacteria PbpA and PbpB) are monofunctional DD-transpeptidases (Dasgupta *et al*. 2006; Plocinski *et al*. 2011). The glycosyltransferase domain catalyses the transfer of the terminal *N*‐glycolylmuramic acid residue from a nascent glycan chain to *N*‐acetylglucosamine of lipid II, releasing decaprenol pyrophosphate (Scheffers and Pinho 2005; van Heijenoort 2007). The C‐terminal DD-transpeptidase domain of PonA1 crosslinks the penultimate d‐Ala to the mDAP of another peptide (Filippova *et al*. 2016). The energy for the DD-transpeptidation reaction stems from the cleavage of the d-Ala-d-Ala peptide bond of the donor peptide resulting in the release of the terminal d-Ala residue (Ghuysen 1991). PonA localises to the poles and septum in mycobacteria and is essential in regulating cell length probably through its interaction with both PknB and the peptidoglycan hydrolase RipA (Hett, Chao and Rubin 2010; Kieser and Rubin 2014). The activities of PonA1 and PonA2 may be altered under different growth conditions as mutants lacking one of the corresponding genes show differential response to cell wall antibiotics (Kieser *et al*. 2015).

The ld-cross-link was first identified by Wietzerbin in 1974 (Wietzerbin *et al*. 1974). Its physiological significance, however, was revealed only in 2008 when Lavollay and colleagues discovered that the PG of stationary phase *M. tuberculosis* almost exclusively contained ld-cross-links (Lavollay *et al*. 2008). An LD-transpeptidase, Ldt_Mt1_, which catalysed the formation of ld-cross-links, was identified and covalent adduct formation with the β-lactamase drugs imipenem, meropenem and ertapenem was demonstrated through mass spectroscopy (Lavollay *et al*. 2008). Subsequently, a second LD-transpeptidase, Ldt_Mt2_ was identified by Gupta and colleagues, and loss of the *ldt_Mt2_* gene in *M. tuberculosis* was shown to result in altered colony morphology, loss of virulence and increased sensitivity to amoxicillin-clavulanate (Gupta *et al*. 2010). In addition, Ldt_Mt2_ is critical for growth in the absence of PonA1 or PonA2, suggesting PBPs and Ldt_Mt2_ together support new cell wall synthesis during growth (Kieser *et al*. 2015).

The influence of LD-cross-linking on mycobacterial physiology and drug susceptibility was further investigated by Baranowski and colleagues, making use of an *M. smegmatis* strain lacking Ldts as a model (Baranowski *et al*. 2018). It was observed that in the absence of LD-cross-links, the rigidity of aging cell wall was compromised leading to the formation of spherical blebs. The increased susceptibility of *M. tuberculosis* to DD-transpeptidase targeting β-lactams was also confirmed and it was demonstrated that inhibiting both DD- and LD-transpeptidases with amoxicillin and meropenem respectively, resulted in synergistic lowering of the MIC (Baranowski *et al*. 2018).

The two-step reaction of LD-transpeptidases begins with the acylation of the enzyme by the third residue (l-chiral centre) of the tetrapeptide donor, followed by deacylation of this acyl-enzyme intermediate by the third residue (d-chiral centre) of the adjacent acceptor stem resulting in the formation of a LD-peptide linkage. The crystal structure of the extra-membrane domain of Ldt_Mt2_ with a bound PG fragment (Erdemli *et al*. 2012) showed that each monomer consists of two globular domains: an N-terminal domain resembling an immunoglobulin domain, and a C-terminal catalytic domain, consisting of a β sandwich with two mixed β sheets characteristic of the YkuD fold (Bielnicki *et al*. 2006), with the two domains connected by a short linker molecule. Structural, kinetic and calorimetric data revealed a catalytic mechanism for Ldt_Mt2_ in which both acyl-acceptor and acyl-donor substrates enter from the same side to reach the catalytic site (Both *et al*. 2013; Triboulet *et al*. 2015; Bhattacharjee *et al*. 2019).

The PG layer was long regarded as a network with mainly DD-cross-links. However, the type of cross-links in PG can switch to mainly LD-cross-links in response to changes in the environment and growth phase in Mycobacteria (Gupta *et al*. 2010) and *E. coli* (Hugonnet *et al*. 2016). The shift to LD-cross-links allows cells to grow in the presence of most β-lactam antibiotics that target PBPs and not LD-transpeptidases, and the use of tetrapeptide donors by LD-transpeptidases allows for the generation of peptide cross-links in the absence of *de novo* synthesis. These features might facilitate the repair of damaged PG (Morè *et al*. 2019) or render PG resistant to hydrolytic enzymes (Lavollay *et al*. 2008), which could be vital during stationary phase or persistence.

PG transpeptidases are verified but difficult targets for antimicrobial drug discovery as (i) due to their redundancy, many of these enzymes can substitute the function of another and (ii) the drugs most commonly used to target them, the β-lactams, are prone to inactivation by β-lactamases.

Drug development efforts targeting the LD-transpeptidases have primarily focussed on further exploring the potential of carbapenems, particularly meropenem, as Ldt inhibitors. Shortly after the identification of Ldt_Mt1_, Hugonnet and colleagues demonstrated that a combination of meropenem and clavulanate was able to kill 13 different XDR strains of *M. tuberculosis* with MIC values > 1 µg/mL (with one exception at 1.25 µg/mL) (Hugonnet *et al*. 2009). This built upon previous work demonstrating that clavulanate can act as an irreversible inhibitor of the *M. tuberculosis* β-lactamase BlaC (Hugonnet and Blanchard 2007).

The direct targeting of Ldt_Mt1_ by carbapenems and cephalosporins was investigated by Dubee and colleagues and it was demonstrated that while both classes of β-lactamase are able to form adducts with Ldt_Mt1_, cephalosporins do so 7- to 1000-times slower than carbapenems (Dubee *et al*. 2012). Since then, Kim *et al*. showed that meropenem acts as a suicide inhibitor of Ldt_Mt2_ by inducing conformational changes in the protein; further informing drug design groups about inhibition mechanisms for the protein (Kim *et al*. 2013).

Recently, in a small retrospective observational case study, Paven and colleagues reported that of 18 patients with XDR-TB who were treated with meropenem-clavulanate, 15 had a ‘successful outcome’ (5 ‘cured’ and 10 ‘completed treatment’), while two patients failed to complete the treatment and one died (Payen *et al*. 2018). It is also notable that no adverse effects were observed as a result of the treatment, suggesting that with further study this may present a promising route to new β-lactamase-based TB treatment. However, treatment of *Mycobacterium bovis* BCG with meropenem was also reported to cause a significant increase in endogenous ATP levels and suppression of this ATP spike using drugs such as Bedaquiline attenuated the activity of the former (Shetty and Dick 2018). Therefore, the inclusion of new drugs into combination therapy poses complications of antagonistic activity.

### PG biosynthesis: orientation of mature PG

The orientation of PG has been a subject of debate and speculation throughout the past decades. The classical depiction represents PG glycan strands as parallel to the bacterial membrane surface as this provides better flexibility to accommodate cell division (Koch 1998, Koch 2000; Vollmer and Holtje 2004; Scheffers and Pinho 2005; Vollmer 2008), however, others postulate that PG is arranged perpendicular/orthogonal to the membrane (Pink *et al*. 2000; Dmitriev *et al*. 2003; Dmitriev, Toukach and Ehlers 2005; Meroueh *et al*. 2006). Using atomic force microscopy the glycan strands of *E. coli* were seen running approximately along the short axis of the cell, and the PG was more ordered in rod shaped *E. coli* sacculi than spheroid shaped sacculi from mutant strains (Turner *et al*. 2018). Our current understanding of the order of PG glycan strands is largely derived from studies on rod-shaped *E. coli* and *B. subtilis*, where new glycan strands are inserted circumferentially forming new PG laterally along the side wall with the help of a protein complex called elongasome (Dominguez-Escobar *et al*. 2011; Garner *et al*. 2011; Hussain *et al*. 2018). Whether a similar order of PG glycan strands is present in mycobacteria is not known, as there are key differences between mycobacterial PG biosynthesis and other rod-shaped bacterial PG structure such as the incorporation of nascent PG at polar ends, the presence of a high proportion of LD-cross-linkages and the absence of MreB. The complexity of the mycobacterial cell wall structure, lack of pure samples and the presence of abundant secondary cell wall polymers make it challenging to determine its architecture.

### PG biosynthesis: the *dcw* gene cluster

**Figure 4.**
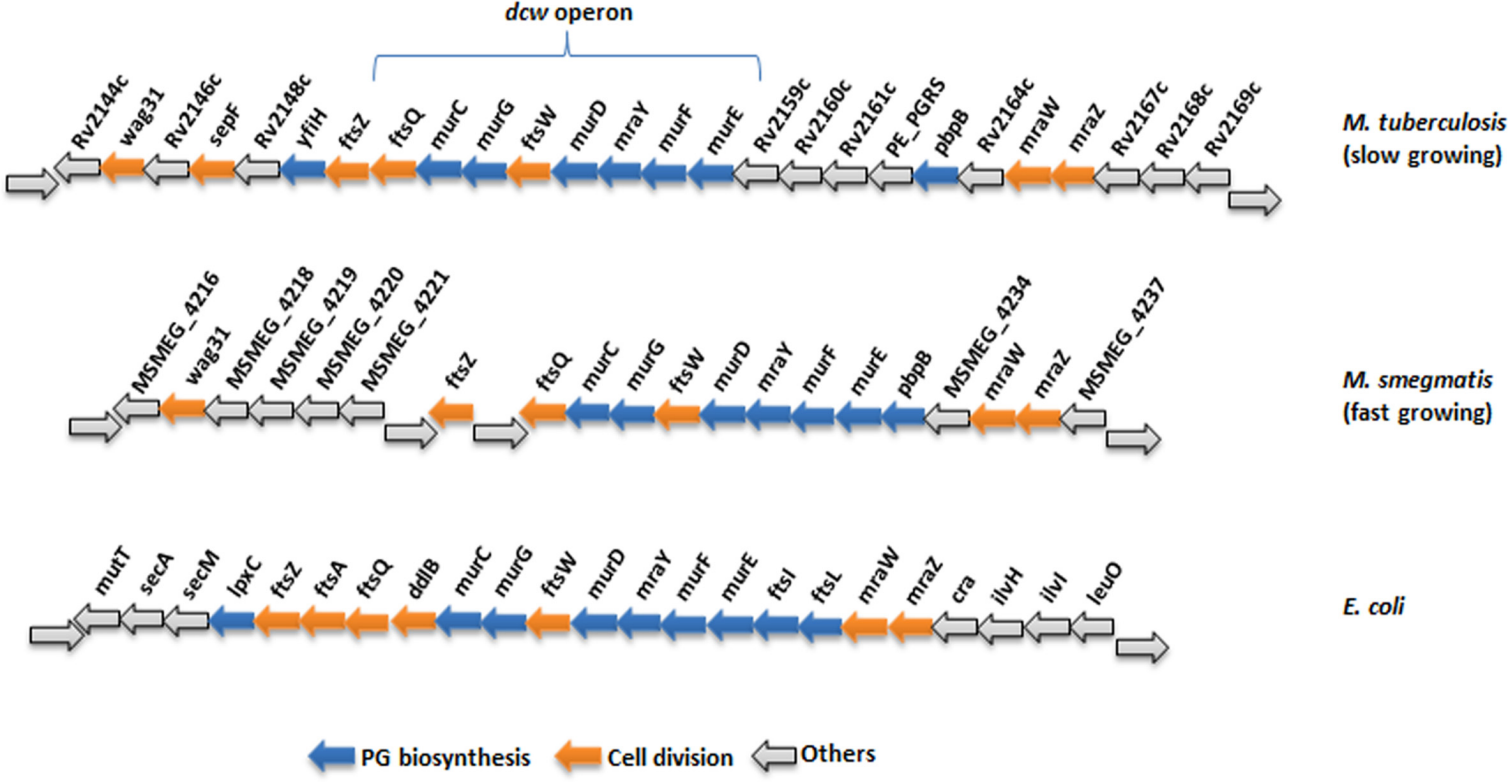
Conservation in genomic organization of *dcw* gene clusters. Here, we compare the differences in the gene content of the *dcw* clusters of fast and slow growing mycobacteria with that of *E. coli*.

Although most of the genes involved in PG biosynthesis have been identified in *E. coli* and their mycobacterial orthologues are known, the differences in the structure of PG between the organisms, however, warrants further investigation into the products of these key genes. Since the sequencing the genome of *M. tuberculosis* (Cole *et al*. 1998), a plethora of information is available about the pathogen, aiding a more targeted approach towards drug discovery and development. The majority of PG biosynthetic genes in *M. tuberculosis* are located on the division-cell wall (*dcw)* cluster along with genes required for cell division (Munshi *et al*. 2013). This clustering of genes is also observed in *E. coli* (Eveland, Pompliano and Anderson 1997), with the exception of the *Rv2161c-Rv2160c-Rv2159c* region and the homologues present only in the slow growing mycobacterial species such as *M. tuberculosis* (Fig. 4). The clustering of cell division genes (*ftsZ, ftsQ* and *ftsW*) and PG biosynthesis genes in the *dcw* operon are indicative of a complex and spatio-temporal coordination between cell division proteins and cell wall precursor synthesis enzymes (Mingorance, Tamames and Vicente 2004; Vicente *et al*. 2006; Munshi *et al*. 2013; Carel *et al*. 2014).

### Degradation and remodelling of PG

PG is a dynamic structure that is constantly synthesised and remodelled. Cell growth, division and septation require the co-ordination of the enzymes required for both PG synthesis and hydrolysis. PG hydrolases (PGH) form a vast group of enzymes including glycosidases, amidases, endopeptidases and carboxypeptidases, which together are capable of fully digesting the PG polymer into soluble fragments. They can act by cleaving bonds within polymeric or soluble fragments of PG (Loessner *et al*. 2002; Vollmer *et al*. 2008), and hence may have a periplasmic, membrane-associated or cytoplasmic location (Holtje 1998). Often called autolysins, they are capable of lysing whole cells when deregulated. Due to their selective ability to cleave PG, the hydrolases have been implicated in a plethora of cellular processes including cell growth, PG maturation, PG turnover, cell division, sporulation, resuscitation and pathogenicity (Goodell and Schwarz 1985; Heidrich *et al*. 2002; Morlot *et al*. 2010; Lee and Huang 2013). Due to their central role in the processes outlined above these enzymes offer a unique opportunity to alter the permeability of the mycobacterial cell wall and so make *M. tuberculosis* and related pathogens more susceptible to drug treatment. In support of their importance in regulation of bacterial physiology Machowski *et al*. recently identified multiple homologues of these PG remodelling genes in *M. tuberculosis* (Machowski *et al*. 2014).

Glycosidases such as *N*-acetyl-β-d-glucosaminidases and *N*-acetyl-β-d-muramidases cleave the polysaccharide backbone of PG, while *N*-acetylmuramyl-l-Ala amidases hydrolyse the amide bond between Mur*N*Ac and l-Ala of the stem peptide. Lytic transglycosylases are, by virtue of their enzymatic reaction, not ‘hydrolases’ but they are usually discussed amongst the PG hydrolases. Unlike other *N*-acetyl-β-d-muramidases like lysozyme the lytic transglycosylases catalyse the formation of a 1,6-anhydromuramyl ring by an intramolecular transglycosylation reaction in the Mur*N*Ac residue of the released PG fragment. Different forms of endopeptidases and carboxypeptidases are capable of hydrolyzing amide bonds either in the stem peptide or cross-linkages of PG (Scheurwater, Reid and Clarke 2008; Vollmer *et al*. 2008).

The presence of three major autolysins, *N*-MurGly-l-Ala amidase, an amino peptidase and an endopeptidase in *M. smegmatis*, was first reported in 1977 (Kilburn and Best 1977) followed later by partial characterisation of a β-glycosidase from *M. phlei* (Li *et al*. 1999).

DacB2 from *M. tuberculosis* is a DD-carboxypeptidase (Class C PBP) that removes d-Ala residues at position 5 of pentapeptides (Kumar *et al*. 2012). The overexpression of *dacB2* results in altered morphology and defective biofilm formation, while its deletion leads to increased intracellular survival of the mutant in THP-1 cells (Bourai, Jacobs and Narayanan 2012). DacB2 was also reported to possess dd-endopeptidase activity (Baranowski *et al*. 2018). Thus, it can affect cell morphology by hydrolysing the DD-cross-links as well providing tetrapeptide substrates for LD-transpeptidases by removing the terminal d-Ala residue.

Early investigations by Votyakova (Votyakova *et al*. 1994) and Mukamolova (Mukamolova *et al*. 1998b; Mukamolova *et al*. 1999; Mukamolova *et al*. 2002) in *Micrococcus luteus* suggested the presence of a resuscitation promoting factor (Rpf) with lytic transglycosylase activity responsible for the release of PG fragments (Fig. 2). This ground-breaking discovery led to the identification of five Rpfs (*rpfA*-*rpfE*) in *Mycobacterium* spp. (Cole *et al*. 1998; Mukamolova *et al*. 1998a; Sutcliffe and Harrington 2004), which significantly enhanced our understanding not only of the remodelling of cell wall PG during daughter cell formation, but also the processes behind dormancy and reactivation of bacilli during active and latent TB. Although all five Rpfs are dispensable for mycobacterial growth, the lack of three of the five *rpfs* leads to avirulence in mice and an inability to resuscitate from dormant bacilli (Downing *et al*. 2005). Furthermore, the lack of spontaneous reactivation of the *rpf* deletion mutants reveals a synergy between them, suggesting that their function cannot be substituted by another enzyme (Downing *et al*. 2005, Kana *et al*. 2008). Interestingly, an Rpf-related motif identified in mycobacteriophages is involved in PG degradation to facilitate phage infection during the stationary phase (Piuri and Hatfull 2006). This suggested that the Rpf-related motif was important for the breakdown of PG with extensive ld-cross-links such as those encountered in dormant *M. tuberculosis*. The Rpfs have detectable sequence homology with lysozymes and lytic transglycosylases (Cohen-Gonsaud *et al*. 2004; Mukamolova *et al*. 2006). The C-terminal domain of RfpB from *M. tuberculosis* comprises a PG binding cleft with two parts. One forms a compact lysozyme-like fold resembling that of c-lysozyme, whereas the other resembles soluble lytic transglycosylase proteins such as *E. coli* Slt70 (van Asselt, Thunnissen and Dijkstra 1999; Cohen-Gonsaud *et al*. 2005). Although the catalytic domains in RpfE and RpfC are similar to those in RfpB (Chauviac *et al*. 2014; Mavrici, Prigozhin and Alber 2014), the substrate-binding site in RpfE is more positively charged than that in RpfB. This suggests that these Rpfs either function optimally at different pH values or cleave different micro-domains of PG.

Due to the network-like structure of PG, its enlargement during cell growth and division requires the synergistic activity of both biosynthetic and hydrolytic enzymes for the incorporation of new glycan strands into the growing PG sacculus. PG hydrolases can destroy the integrity of the sacculus; hence their activity must be controlled in the cell to avoid autolysis. There is mounting evidence that the biosynthetic and hydrolytic enzymes interact with each other to form complexes capable of synergistic breaking and making of bonds in PG and regulating activity. In *E. coli*, Slt70 (a soluble lytic transglycosylase) forms a complex with PBP 3 (a PG transpeptidase) and PBP 7/8 (a dd-endopeptidase) and both enzymes synergistically degrade PG (Romeis and Holtje 1994). Slt70 also interacts with PBP1B, PBP1C and PBP 2 (von Rechenberg, Ursinus and Holtje 1996). The lytic transglycosylase MltA interacts with PBP1B (a bifunctional synthase) and MipA (MltA-interacting structural protein). MtlB interacts with PBP1B, PBP1C and PBP 3 (von Rechenberg, Ursinus and Holtje 1996; Vollmer, von Rechenberg and Holtje 1999). In *Mycobacterium* spp. the resuscitation promoting factors RpfB and RpfE interact with the endopeptidase RipA at the septum of dividing bacteria (Hett *et al*. 2007; Hett and Rubin 2008). An interaction between SltB1 and PBP2 has been observed in *P. aeruginosa* (Legaree and Clarke 2008; Nikolaidis *et al*. 2012).

Mycobacterial RipA is a d-Glu-*m*DAP endopeptidase that cleaves between positions 2 and 3 of the stem peptide (Both, Schneider and Schnell 2011). RipA is essential in *M. tuberculosis* (Sassetti, Boyd and Rubin 2003) and its depletion in *M. smegmatis* causes severe growth inhibition (Hett and Rubin 2008). Unregulated, RipA behaves like an autolysin in *M. tuberculosis* and *M. smegmatis*, resulting in spherical cells that eventually lyse (Chao *et al*. 2013). RipA, belonging to NlpC/P60 family, has a Cys-His-Glu catalytic triad residing in the C-terminal catalytic domain, (Ruggiero *et al*. 2010) and is regulated by proteolytic cleavage by MarP (Botella *et al*. 2017a). RipA becomes a lethal autolysin in the absence of protein interactions that are necessary to control its proteolytic activation (Chao *et al*. 2013). RipA-mediated cleavage in PG and its activation is more robust in the fast-growing environmental species *M. smegmatis* compared to the slow growing *M. tuberculosis* or *M. bovis* BCG—presumably a consequence of the varying demands for PG hydrolysis required at differing growth rates. RipB has unique features in its N-terminal segment, which runs across the catalytic core domain and forms a helix instead of the β-hairpin loop seen in the homologous module of RipA (Both, Schneider and Schnell 2011). These enzymes are not essential and so are not attractive drug targets.

*N*-acetylmuramyl-l-Ala amidases hydrolyse the bond between Mur*N*Ac and the peptide portion of the PG and have been implicated in PG degradation and turnover, cell separation, antibiotic resistance and spore formation (Heidrich *et al*. 2001; Vollmer *et al*. 2008). PG amidases fall into two groups; one containing the amidase_2 domain and other the amidase_3 zinc-dependent domain (which includes the *E. coli* AmiA, AmiB and AmiC and *B. subtilis*CwlB and CwlC enzymes). The first autolysin identified in *M. tuberculosis* was an amidase known as CwlM (Rv3915), which has since been established to have a regulatory role in PG synthesis and maintenance as mentioned in preceding sections. Another homologue (Rv3717) of the *E. coli* PG amidases recently identified in *M. tuberculosis* was found to lack a PG-binding domain and is active on muramyl dipeptide, but not on polymerised PG (Kumar *et al*. 2013; Prigozhin *et al*. 2013). The catalytic function identified by the structural analysis of Rv3717 indicates that the catalytic pocket of this enzyme is shaped as a blind tunnel and accommodates only PG monomers, as peptide cross-links in polymerised PG cannot enter the tunnel. It uses an overall positive charge on its surface to bind to its substrate and regulates its activity through an auto-regulatory β-hairpin (Kumar *et al*. 2013). As this is a cytosolic protein the activity of this enzyme depends on the action of another PG hydrolase cleaving the cross-links of PG fragments released from the cell wall and transported back into the cytoplasm. This indicates a possible involvement for Rv3717 in PG recycling (Prigozhin *et al*. 2013).

### Transport and recycling of degraded PG components

A study of turnover rates in *E. coli* led to the serendipitous discovery of PG recycling (Goodell and Schwarz 1985). The extent of PG fragments released from the cell wall of growing bacteria, is in the range of 25%–50% in Gram-positive organisms such as *B. subtilis* (Mauck, Chan and Glaser 1971) and *E. coli* (Chaloupka and Strnadova 1972; Goodell 1985; Goodell and Schwarz 1985). However, *E. coli* recycles about 90% of the turnover products, releasing only 0%–5% of the PG material into the culture supernatant. Until recently, it was believed that PG recycling is a characteristic of Gram-negative bacteria only (Herve *et al*. 2007). However, the identification of orthologues of recycling enzymes in *B. subtilis* and subsequent muropeptide recovery studies have uncovered evidence of PG recycling in Gram-positive organisms as well (Reith and Mayer 2011; Kluj *et al*. 2018). Apart from the obvious advantages associated with conservation of resources and energy, PG recycling also plays a significant role in monitoring the integrity of the cell wall, cell signalling and antibiotic resistance *via* β-lactamase induction (Jacobs *et al*. 1994). Additionally PG recycling plays a crucial role in the suppression of the innate immune response, by effectively recovering cell wall muropeptides that would otherwise stimulate immunity, *via* proteins that recognise PG (Boudreau, Fisher and Mobashery 2012; Johnson, Fisher and Mobashery 2013).

Although there is little knowledge about PG recycling in *M. tuberculosis*, some similarities have been observed with the well-studied system in *E. coli*. Recycling of PG precursors entails lateral wall and septal PG degradation to form Glc*N*Ac-(1→4)-1,6-anhydro-Mur*N*Ac-peptide monomers, Glc*N*Ac-(1→4)-1,6-anhydro-Mur*N*Ac dissacharides and free peptides (Goodell 1985). Pulse-chase experiments with tritiated *m*DAP provided evidence to suggest that many of the cell wall precursors were recycled (Goodell and Schwarz 1985). AmpG from *E. coli*, also required to induce AmpC (a β-lactamase), was identified as the permease transporting the primary products of the action of lytic transglycosylases on PG into the cytoplasm (Korfmann and Sanders 1989; Jacobs *et al*. 1994; Cheng and Park 2002). Although Gram-positive organisms lack an AmpG homologue, the presence of muropeptides with various length peptide side chains (tetrapeptides, dipeptides) (Mahapatra *et al*. 2008), as well as inducible β-lactamases (Flores, Parsons and Pavelka 2005), suggests that an equivalent pathway for the recycling pathway for PG exists in mycobacteria. Once the muropeptides have been transported back to the cytoplasm, various amidases and epimerases, which are non-essential for growth, further process them to products that can be utilised for *de novo* PG precursor synthesis (Park and Uehara 2008).

Homologues of the enzymes involved in the import, breakdown and recycling of muropeptide in the cytoplasm are yet to be identified in *M. tuberculosis*. In the preceding section we discussed the various enzymes involved in degradation and remodelling of the cell wall PG. Recent work by Moynihan *et al*. provided evidence for the uptake of muropeptide fragments in *M. tuberculosis* and *M. bovis* BCG; where they show cleavage of stem peptide followed by disaccharide cleavage by LpqI (NagZ) and finally lactyl-ether removal from the Mur*N*Ac moiety (Moynihan *et al*.^2019^). NagA is involved in PG recycling through the deacetylation of Glc*N*Ac-6P in a number of other organisms, including *E. coli, B. subtilis, S. aureus* and *Streptomyces coelicolor*. A putative *nagA* gene in an operon distinct from other bacterial organisms, has been identified in *M. tuberculosis* (Ahangar *et al*. 2018). *Mycobacterium tuberculosis nagA* is essential and encoded along with genes involved in PG biosynthesis and carbohydrate uptake, indicating that it may play a role in the recycling of Glc*N*Ac6P derived from the breakdown of PG.

Structural characterisation of NagA reveal it to be a dimer, with each unit organised in two domains with active site pockets occurring in domain I (Ahangar *et al*. 2018). The crystal structure reveals the binding of the Glc*N*Ac-6P substrate to *M. smegmatis* NagA and indicates that the active site does not undergo any major structural change on binding the substrate. However, two loop regions demonstrate a capacity for conformational flexibility and an importance in facilitation of substrate binding, providing opportunity for designing inhibitor molecules (Ahangar *et al*. 2018). Biochemical characterization shows a clear preference of NagA for Glc*N*Ac6P over other amino sugar analogues. The stringent recognition of this fragment is hypothesised to ensure the integrity of PG recycling (Ahangar *et al*. 2018).

The essentiality of *nagA* makes it a feasible drug target. Eukaryotic homologues of the enzyme have low sequence identity and accommodate larger, glycolyl substituted substrates. This property makes the two homologues distinct, however, small inhibitors acting on *M. tuberculosis* NagA may be accepted by mammalian homologues thereby giving rise to cytotoxicity (Bergfeld *et al*. 2012).

Recycling of stem peptides is facilitated by murein peptide ligase (Mpl) in Gram-negative organisms. Mpl ligates di-, tri- and tetrapeptide stems onto UDP-Mur*N*Ac in an ATP-dependent step thereby replacing the functions of MurC-F playing an important role in the transition of cell towards stationary phase, as observed in *E. coli* (Talukder *et al*. 1996). An *mpl* homologue has not been identified in Gram positive bacteria or in any mycobacterial species.

### Role of PG in cell signalling

**Figure 5.**
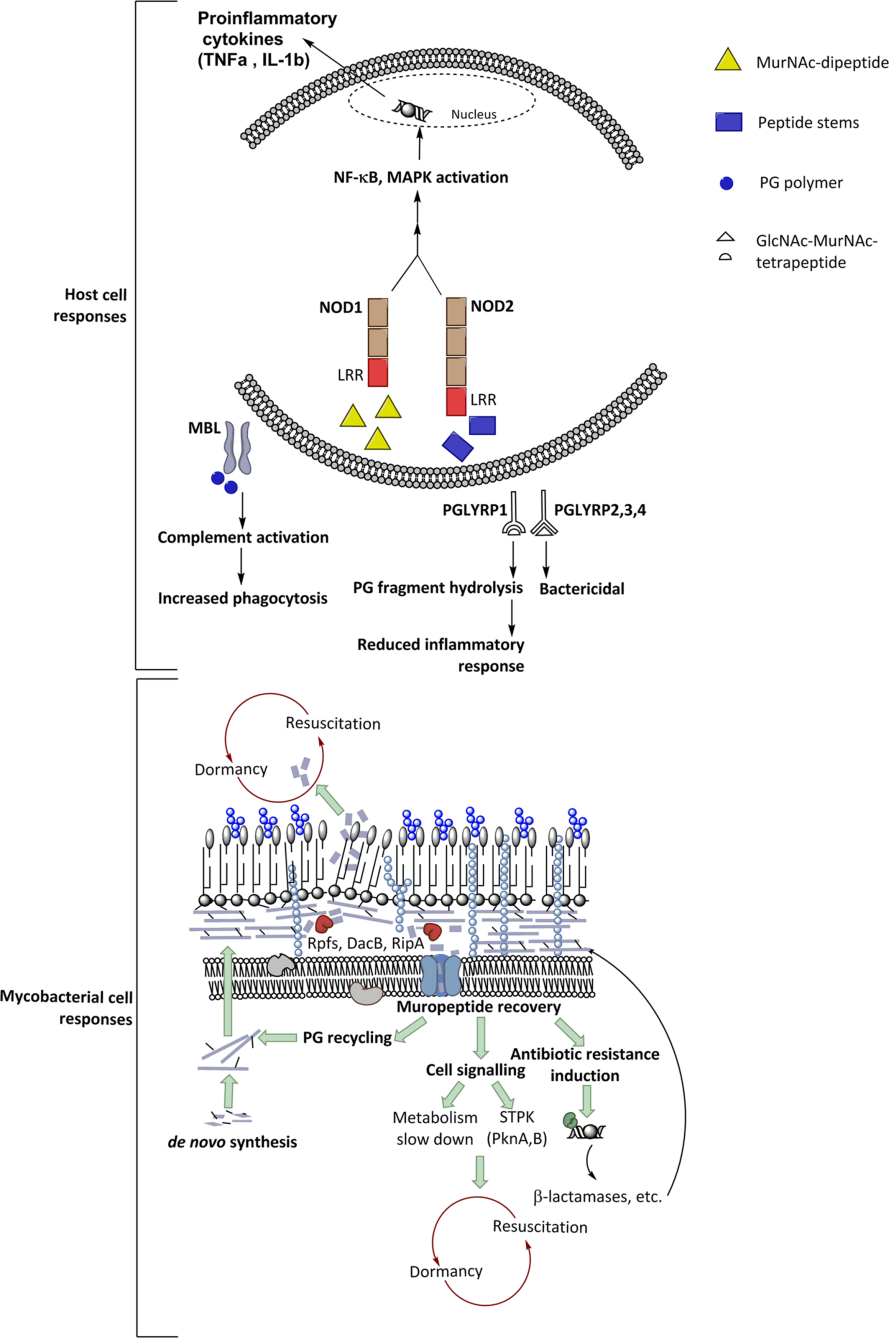
The role of PG and its breakdown products in signalling. The digestion of PG by hydrolases generates products like muramyl dipeptide and muramyl tripeptide that are key signalling molecules to resuscitate bacterial cells and may induce β-lactamase. PG fragments are encountered by the host immune surveillance systems either extracellularly (e.g. in serum) or are phagocytosed triggering various pro-inflammatory host immune responses.

PG fragments released from the cell envelope during growth and cell division play important roles in cellular signalling and inter-bacterial communication (Humann and Lenz 2009) (Fig. 5). Rpfs produce PG fragments that lead to the resuscitation of dormant mycobacteria (Mukamolova *et al*. 1998a; Mukamolova *et al*. 1999; Nikitushkin *et al*. 2013). RpfC and RpfD are restricted to pathogenic and environmental mycobacteria and are secreted into the culture medium, unlike RpfA, RpfB and RpfE, which are membrane-bound (Machowski *et al*. 2014). This implies that specific PG products are likely to act as messengers, which can act as a signal to reawaken dormant cells (Mukamolova *et al*. 1998a; Zhang *et al*. 2001; Machowski *et al*. 2014). The release of *m*DAP-containing muropeptides during growth triggers sporulation in *B. subtilis* and by binding of the serine threonine protein kinase (STPK) PrkC to the PG fragment (Shah *et al*. 2008). It is possible that in mycobacteria the PG fragments released by Rpfs bind PknB, triggering a cascade of signals leading to resuscitation of the bacteria (Fig. 5). Furthermore, Russell-Golman *et al*. also showed that mycobacterial double mutants of RpfA and RpfE show a reactivation-deficient and attenuated phenotype (Russell-Goldman *et al*. 2008), suggesting that Rpfs serve as virulence factors (Romano *et al*. 2012). In addition, Rpfs induce elevated levels of cytokines in cultures from individuals with latent TB (Riano *et al*. 2012).

Although PG has been the subject of intense study over the last number of decades, the changes that occur when a cell enters and exits stationary phase are not well understood. In some bacteria, d-amino acids are incorporated into the PG polymer in the stationary phase, which affects the amount of PG per cell (Lam *et al*. 2009; Cava *et al*. 2011). d-Ala inhibits the germination of spores in *B. subtilis* (Chesnokova *et al*. 2009). d-Met and d-Leu were the most prominent non-canonical d-amino acids incorporated into the PG during the stationary phase in *Vibrio cholerae* (Lam *et al*. 2009).

The ability of a host to recognise bacterial components and trigger an innate immune response is the first line of defence against infection. The fact that eukaryotes do not produce PG has led to the evolution of several strategies to detect PG or PG fragments (Humann and Lenz 2009; Otten *et al*. 2018). The germline-encoded pattern-recognition receptors (PRRs) are expressed mainly on immune cells and recognise pathogen-associated molecular patterns (PAMPs) and mediate the production of immunoregulatory cytokines such as tumour necrosis factor (TNF) and type I interferons (IFNs) (Medzhitov and Janeway 1997). Toll-like receptors (TLR) are activated by immunomodulatory lipoproteins in mycobacteria such as lipomannan (LM), lipoarabinomannan (LAM), mannosyl capped LAM (ManLAM) or phosphoinositide capped LAM (PILAM) and PG components present on the cell surface or endosome/lysosome membranes (Gilleron, Quesniaux and Puzo 2003; Quesniaux *et al*. 2004; Singh *et al*. 2012; Esin *et al*. 2013; Xie *et al*. 2013). The cytoplasm of the cell is also under scrutiny for PAMPs that breach the cell membrane. These PAMPs are recognised by constitutively expressed Nod-like receptors (NLR) like NOD, NALP and NAIP. They consist of the NACHT or NOD oligomerisation domain and a motif similar to the microbial sensing leucine-rich repeat (LRR) receptors present in the TLRs. While NOD1 specifically recognises the γ-d-glutamyl-*m*DAP peptide of PG fragments, NOD2 and NALP3 are activated by intracellular muramyl dipeptides (Mukamolova *et al*. 1998a; Zhang *et al*. 2001; Chamaillard *et al*. 2003a; Girardin *et al*. 2003a; Chamaillard *et al*. 2003b; Uehara *et al*. 2006; Humann and Lenz 2009; Pandey *et al*. 2009; Killick *et al*. 2013; Machowski *et al*. 2014). Thus, any changes in the structure of PG could impair the innate immune responses of the host.

### Targeting PG biosynthesis to tackle antibiotic resistance

^(R)^The ‘Golden Age’ of antibacterial drug discovery appears to be over: since the 1990s there has been a void in the development of new drugs for the treatment of bacterial infections (Silver 2011). More specifically, for TB the current frontline treatment regimen is a cocktail of four drugs (isoniazid, rifampicin, pyrazinamide and ethambutol) that was first introduced in the 1960’s (Zumla, Nahid and Cole 2013). The emergence of MDR- and XDR-TB strains, however, have reinvigorated the search for antimycobacterial agents with novel modes of action (Young *et al*. 2008). Recent progress in this area includes the approval of Bedaquiline (Sirturo) (Mahajan 2013; Koul *et al*. 2014) and Delamanid (Deltyba) (Ryan and Lo 2014).

Pathways involved in the biosynthesis of cell wall PG have historically been recognised as important drug targets. This is demonstrated by the fact that a significant number of the antibiotics in current clinical use, primarily β-lactams (e.g. penicillins and cephalosporins) and glycopeptides, act by inhibiting the later stages of PG biosynthesis (Fig. 2). *Mycobaterium tuberculosis* is considered resistant to many β-lactams, due to a combination of the following factors: the thick, complex and hydrophobic nature of its cell envelope, the presence of one or more active β-lactamases (Nampoothiri *et al*. 2008) and low-affinity PBPs (Chambers *et al*. 1995). A number of clinically useful TB drugs, however, do target cell wall processes. These include: (i) the first line anti-TB drug isoniazid which inhibits MA biosynthesis (Zhang *et al*. 1992), (ii) ethambutol, another frontline drug, which inhibits AG synthesis (Mikusova *et al*. 1995) and (iii) a second line drug d-cycloserine which is a broad spectrum antibiotic that inhibits PG biosynthesis *via* inhibition of Alr and Ddl (Zhang 2005). Delamanid, like isoniazid, also targets genes in MA biosynthesis (Ryan and Lo 2014); and Pretomanid (PA-824), an antitubercular drug under regulatory review by the FDA, has also been shown to affect not only respiratory genes, but also transcription of genes responsive to known cell wall inhibitors like isoniazid (Manjunatha, Boshoff and Barry 2009). The efficacy of these drugs highlights the essentiality of the mycobacterial cell wall PG and highlights the opportunity to develop novel chemical entities, which target enzymes involved in the synthesis of the mycobacterial cell envelope (Feng and Barletta 2003; Silver 2006; Prosser and de Carvalho 2013). Further research to identify the unique features of the mycobacterial PG enzymes with respect to their structure, function and regulation can advance the development of narrow-spectrum, targeted antibiotics.

**Figure 6.**
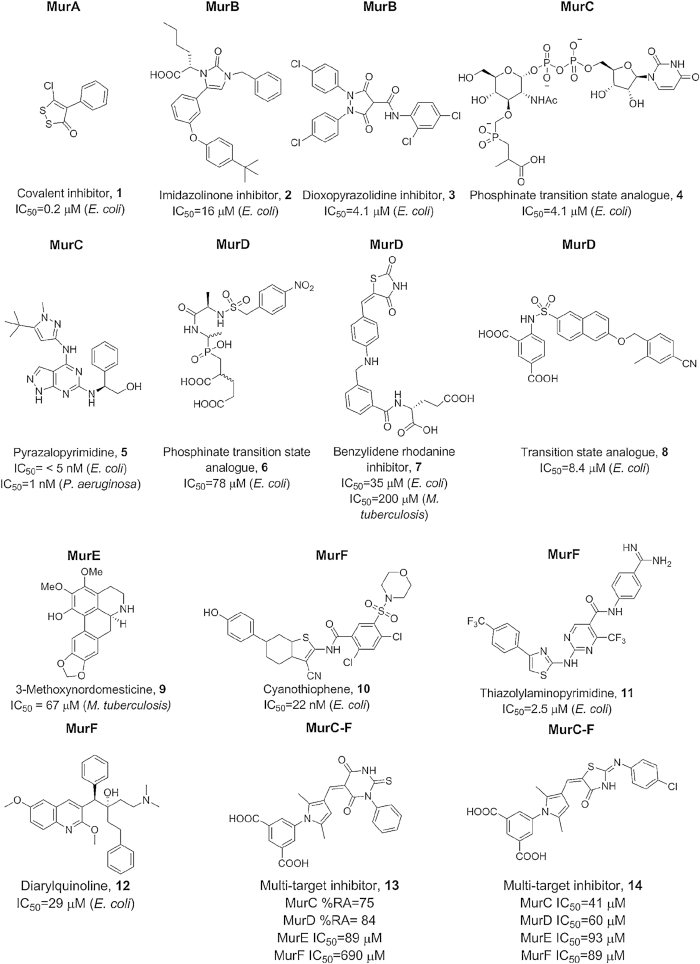
Structures of representative inhibitors of MurA-F with inhibitory data.

To date the early stages of PG biosynthesis have been poorly exploited as antibacterial targets, and so present a significant opportunity for the development of the next generation of antimycobacterial drugs. In particular, the enzymes involved in the cytoplasmic stage of the PG biosynthetic pathway are currently considered as new targets for drug discovery. This interest, however, has yet to translate to the development of a clinically useful molecule. Although inhibitors against MurA and MurB have been reported previously (Baum *et al*. 2001; Bronson *et al*. 2003; Yang *et al*.^2006^) (**1**, **2**, **3**, Fig. 6.) herein, we will review the progress made to date towards the development of inhibitors targeting the ATP-dependent Mur ligases MurC–F involved in the cytoplasmic phase of PG biosynthesis.

The Mur ligases are found in all clinically significant bacterial pathogens, and so inhibitors would be expected to be broad spectrum. In addition, (i) there are conserved structural and sequence motifs and 3 binding sites in MurC-F against which antimicrobial agents can be designed (e.g. MurE Fig. 3) and (ii) partial inhibition at multiple steps of PG synthesis (various Mur enzymes) compared to inhibition of a single enzyme in the pathway may provide a more efficient route to obtaining bactericidal activity and decrease the likelihood of development of resistance. As a result, pan or multi-target inhibitors are conceivable and these may provide a better approach to obtaining robust antibacterial activity.

Adding to the appeal of the ATP-dependent Mur ligases as targets for drug design, many X-ray crystal structures have been solved for proteins from different bacterial species (Bertrand *et al*. 1999; Deva *et al*. 2006; Basavannacharya *et al*. 2010a). However, only the X-ray crystal structure of MurE from *M. tuberculosis* is currently available (Basavannacharya *et al*. 2010a; Basavannacharya *et al*. 2010b). Homology models of MurC and MurD from *M. tuberculosis* and MurC-F from *M. leprae* have been built based on sequence identification with the corresponding homologue from either *E. coli* or *H. influenzae* (Anuradha *et al*. 2010; Arvind *et al*. 2012; Shanmugam, Anbazhagan and Natarajan 2012); Shanmugam and Natarajan 2012). Docking of native substrates to these models aligned well with known X-ray crystal structures and good homology was observed between conserved binding site residues (Anuradha *et al*. 2010; Arvind *et al*. 2012; Shanmugam and Natarajan 2012). *In silico* docking of naphthalene sulphonamide inhibitors recapitulated the binding mode observed in X-ray crystal structures and highlighted the importance of the Glu moiety as a driver of affinity for MurD (Arvind *et al*. 2012). One may expect that inhibitors of Mur ligases from other species may also inhibit the mycobacterial enzymes. These models, therefore, provide a good starting point for the design of novel ligands, however, experimental validation of the predicted structures is currently lacking.

The progress made to date in the design of Mur ligase inhibitors has recently been reviewed, although it is noteworthy that activity data on *M. tuberculosis* are woefully missing (Silver 2011; Perdih *et al*. 2014; Sangshetti *et al*. 2017). The inhibitors discovered to date fall primarily into three classes: (i) transition state analogues, which contain for example phosphinate or sulphonamide functional groups which mimic the tetrahedral transition state formed during the enzyme-catalysed reaction (Fig.   3) (Marmor *et al*. 2001; Reck *et al*. 2001), (ii) phosphate/diphosphate mimics which typically contain a heterocyclic moiety (e.g. rhodanine, thiazolidindione) (Barreteau *et al*. 2012) and (iii) inhibitors that bind to the ATP binding site (Hameed P *et al*. 2014). A number of core functional groups are repeatedly found in inhibitors of MurA to F, particularly those which act as mimics of the tetrahedral transition state or are phosphate bioisosteres. The majority of inhibitors developed to date have arisen from HTS ( *in vitro* or *in silico*) followed by structure-guided design, however, natural products have also been the source of a number of inhibitors (Guzman *et al*. 2010; Guzman *et al*. 2013b).

The majority of published MurC inhibitors are either phosphinate transition state analogues (**4**, Fig. 6) (Marmor *et al*. 2001; Reck *et al*. 2001) or l-Ala analogues (El Zoeiby *et al*. 2003). To the best of our knowledge, however, antibacterial activity has not been demonstrated for either class, which is perhaps unsurprising as the above compounds are very polar and are unlikely to be membrane permeable. The most promising MurC inhibitors identified to date were reported in a study by Astra Zeneca in which a series of potent pyrazolopyrimidines were developed (Fig. 6, **5**). Compound **5** was proposed to bind into the ATP binding site and encouragingly, also demonstrated bactericidal activity, albeit against efflux deficient mutants (MIC = 3.1 and 1.5 µM against *E. coli* and *P. aeruginosa* respectively). Importantly, **5** also demonstrated selectivity by not inhibiting a panel of human kinases. From mutation resistance maps **5** was suggested to be selective for MurC over the other Mur ligases, however, biochemical data to test for inhibition of MurD-F is lacking. Most significantly, the mode of action of the pyrazolopyrimidines was conclusively demonstrated to be as a result of inhibition of MurC. This is a particularly noteworthy advance, as it is the first example in which an antibacterial activity of a Mur ligase inhibitor has been demonstrated to be due to target engagement (Hameed P *et al*. 2014).

MurC phosphinate transition state analogues are also potent, sub-nanomolar inhibitors of MurD. Replacement of the uridine core with an aryl sulphonamide as a phosphate mimic afforded the simplified inhibitor **6** (Fig. 6), which retained an IC_50_ in the low micromolar range against MurD and was more amenable to optimisation by medicinal chemistry (Strancar *et al*.^2006^). The d-glutamate moiety or conformationally restrained analogues are present in the majority of published inhibitors, as they are key to potent inhibition of MurD (**7**, **8**, Fig. 6.) (Barreteau *et al*. 2012). Another class of MurD inhibitors feature a benzylidene rhodanine moiety, which occupies the uridine diphosphate binding site (**7**, Fig. 6). Although **7** is a low micromolar inhibitor of the *E. coli* enzyme, it inhibits the mycobacterial enzyme with an IC_50_ of 200 µM, highlighting the challenges associated with the design of broad spectrum agents (Barreteau *et al*. 2012).

A number of the reported MurD inhibitors also inhibit MurE and, again, contain phosphinate groups (or sulphonamides) that mimic the tetrahedral intermediate formed during the enzyme-catalysed reaction (Tanner *et al*. 1996; Strancar *et al*. 2007). Alternative scaffolds have been identified from natural product sources and are of particular interest here due to their demonstrated antimycobacterial activity. For example *N*-methyl-2-alkenyl-4-quinolone derivatives isolated from Evodia rutaecarpaand aporphine alkaloids isolated from *Ocotea macrophylla* inhibit the activity of MurE from *M. tuberculosis* (**9**, Fig. 6,) and prevent cell growth at micromolar concentrations (Guzman *et al*. 2010; Guzman *et al*. 2011; Wube *et al*. 2011; Guzman *et al*. 2013b). These natural products also inhibit the growth of a number of other Gram-positive and Gram-negative bacteria, but at much higher concentrations than those required against mycobacteria. It has yet to be demonstrated that the observed antibacterial activity is due to inhibition of MurE only, however, given the relatively low affinity and lipophilicity of these compounds it is unlikely that they are highly selective for their target. The natural product *S*-leucoxine from *Rhodostemonodaphne crenaticupula* and related synthetic 1-tetrahydroquinolines have also been demonstrated to inhibit MurE and mycobacterial cell growth, further investigation, however suggested a pleiotropic mechanism of action (Pesnot *et al*. 2011; Guzman *et al*. 2015).

A significant number of potent nanomolar inhibitors have been developed for MurF. Abbott Laboratories, for example, developed a series of cyanothiophene ligands with micromolar to nanomolar affinity (**10**, Fig. 6) (Gu *et al*. 2004; Stamper *et al*. 2006) and Johnson & Johnson developed a series of potent thiazolylaminopyrimidine containing inhibitors (**11**, Fig. 6) (Baum *et al*. 2006). However, none of the compounds tested demonstrated any significant antibacterial activity. Finally, the identification of diarylquinolines as low micromolar inhibitors of MurF led to the development of **12** (Fig. 6), which exhibited an MIC of 8 µg/mL against a number of bacterial species (*Enterococcus faecalis*, *E. faecium* and *S. aureus*). Compound **12** binds MurF in the presence of ATP and so is likely to occupy the substrate binding site. In addition, treatment of LPS deficient *E. coli* with **12** resulted in the accumulation of the substrate of MurF, however, over expression of MurF did not significantly affect the MIC. Hence, although the diarlyquinolines represent a promising start point for the development of novel antibacterial agents, the mechanism of action warrants further examination.

**Figure 7.**
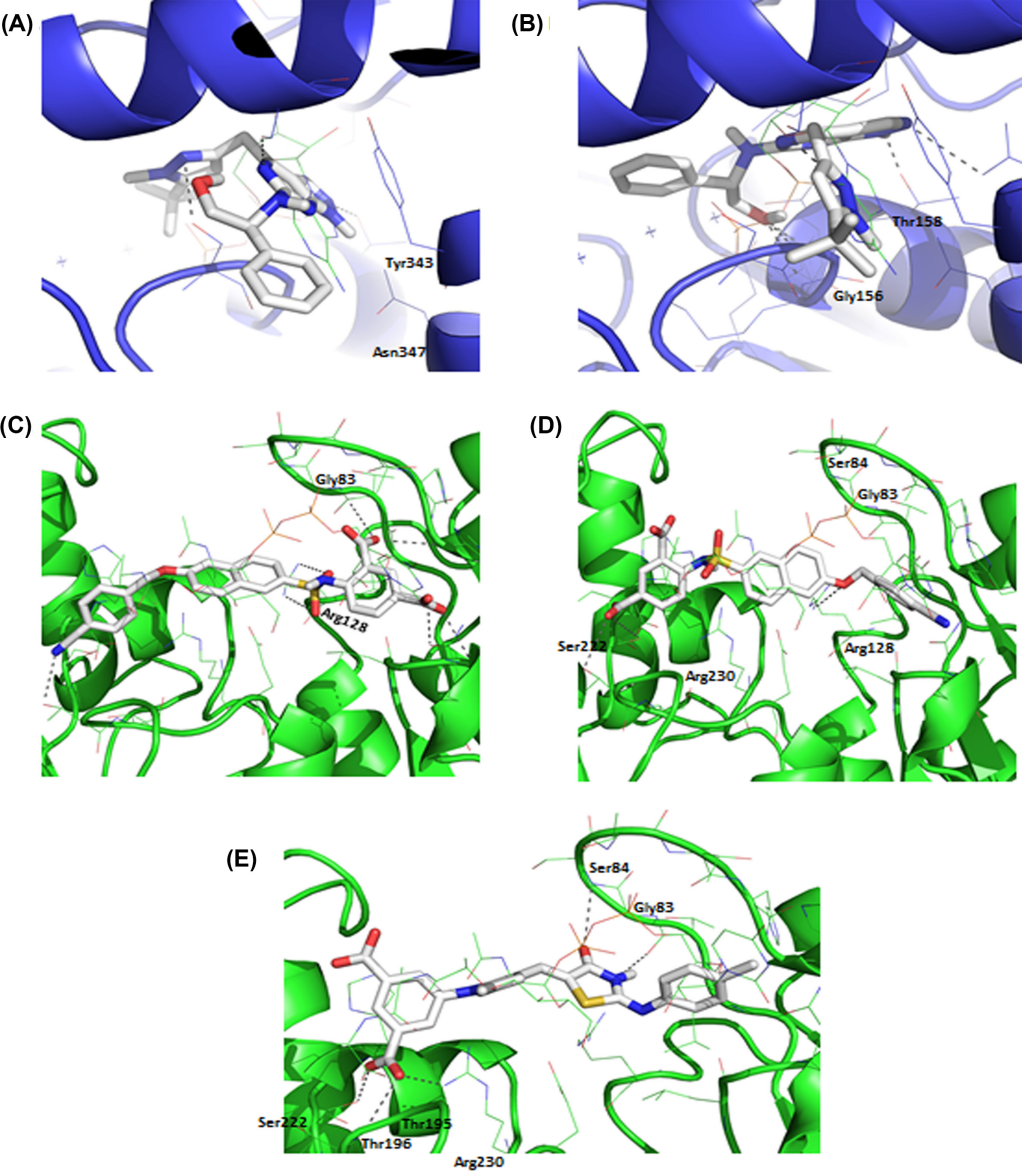
Representative predictive binding pockets for selected compounds to MurE (PDB code 2XJA) from *M. tuberculosis* (overlaid with ATP or UAG as appropriate, shown in lines) **(A)**, Compound 5, predicted binding affinity −9.2 kcal/mol **(B)**, Compound 5, predicted binding affinity −8.9 kcal/mol **(C)**, Compound 8, predicted binding affinity -7.8 kcal/mol (D) Compound 8, predicted binding affinity −7.2 kcal/mol (E) Compound 14, predicted binding affinity −6.8 kcal/mol.

Strategies for the development of multi-target inhibitors of the Mur ligases fall in to two categories: (i) ligands that mimic the shared tetrahedral intermediate of the Mur ligases and (ii) ligands designed to target the ATP-binding site, which has the greatest sequence and structural homology amongst the enzymes. Alternatively, for dual target inhibitors, the substrate binding sites of MurC/D and MurE/F are likely to have sufficient similarity to allow the design of dual inhibitors. The majority of efforts to date have focussed on the first strategy leading to benzene-1,3-dicarboxylic acid 2,5-dimethylpyrrole derivatives (Fig.   6, **13** & **14**) (Perdih *et al*. 2014) and napthyl tetronic acids (Mansour *et al*. 2007) as first multi-target lead compounds. To evaluate the potential of these Mur ligase inhibitors as inhibitors of the mycobacterial enzymes, we performed a docking study (Autodock Vina) (Trott and Olson 2010), using a select number of inhibitors and MurE (pdb code: 2XJA) from *M. tuberculosis* as a model system. The pyrazalopyrimidine **5** was designed to inhibit MurC from *E. coli* (Deva *et al*. 2006). Docking of **5** to MurE from *M. tuberculosis* resulted in the prediction of two major binding modes. In the first binding mode there is good overlap between the pyrazolpyrimidine ring and the adenosine ring of ATP (as observed X-ray crystal structure) allowing a *π-π* stacking interaction with Tyr343 (an aromatic residue at this position is highly conserved amongst the Mur ligases); the pyrazole ring is predicted to occupy the phosphate binding site and forms a H-bonding interaction with Thr158 (part of the conserved diphosphate binding motif) (Fig. 7). In the second binding mode the pyrazole ring forms a *π-π* stacking interaction with Tyr343, the pyrazolopyrimidine moiety is predicted to form polar contacts with two key residues (Asn347 and Thr158) and the hydroxyl group interacts with residues in the highly conserved phosphate binding loop (Gly156, Lys157, Thr158) (Fig. 7B). Given the recapitulation of key interactions formed by ATP within the binding site and similarities with the binding mode predicted by Hameed *et al*. it is feasible that **5** and analogues thereof may provide a starting points for the development of inhibitors for the mycobacterial Mur ligases (Hameed P *et al*. 2014).

The benzylidene rhodanine inhibitor **7** was designed as an inhibitor of MurD from *E. coli* (Zidar *et al*. 2010). In the X-ray crystal structure **7** was found to span the substrate binding site with the rhodanine heterocycle acting as a diphosphate mimic. This binding mode is recapitulated when docked into MurE from *M. tuberculosis*, however, because of the larger binding site the rhodanine heterocycle is predicted to bind in the hydrophobic pocket occupied by the uracil moiety of UAG and not to the diphosphate binding site as in *E. coli* MurD.

Transition state analogue, **8**, another inhibitor of MurD, which contains a conformationally restrained Glu analogue and a sulphonamide moiety as a transition state analogue, was also docked to MurE. This inhibitor was predicted to span the substrate binding site and two alternate binding modes were predicted: (i) in which the Glu mimic occupied the uracil binding site and the sulphonamide the diphosphate binding site and (ii) in which the cyanobenzyl functional group occupied the uracil binding site and the sulphonamide and Glu mimic mapped well onto the observed conformation of UDP-Mur*N*ac-peptide in the X-ray crystal structure of MurE (Fig. 7C, D). However, it is unlikely that the sulphonamide moiety will be able to bind in a similar conformation to the tetrahedral transition state due to the larger and more open binding site of MurE in comparision to MurD. It is important to note that the N-terminal domain exhibits the greatest sequence and structural variation i.e. the N-terminal domain in MurC/D is significantly different to that in MurE/F. This domain is also more flexible and undergoes significant conformational changes on ligand binding; this flexibility is required to enable the Mur ligase to accommodate the growing peptide chain of the PG precursor (Basavannacharya *et al*. 2010b). As docking experiments are based solely on and restricted by the structures/conformations available, these need to be integrated with *in vitro* experiments such as enzyme inhibition kinetics to gain confidence in the targeting of inhibitors.

A related set of compounds designed as multi-target Mur ligase inhibitors are compounds **13**(a dual inhibitor of MurD/E) and **14**(an inhibitor of MurC-F). These were docked against MurE from *M. tuberculosis* (Tomasic *et al*. 2010; Basavannacharya *et al*. 2010a; Perdih *et al*. 2014). For **13**, as expected, the dihydopyrimidine moiety was predicted to act as a diphosphate mimic and the phenyl ring to occupy the uracil binding site. The remainder of the inhibitor maps well onto the conformation of UAG observed in the X-ray crystal structure ( **13** is, however, truncated compared to UAG and so does not span the entire binding site). **14**, which is a moderate inhibitor of MurC-F, is predicted to adopt a similar conformation within the substrate binding site with the rhodanine heterocycle again acting as a diphosphate mimic and the chlorophenyl moiety occupying the uracil binding site. The increased flexibility in **14**, compared to its precursor **13**, allows the heterocycle to form polar contacts with key residues in the diphosphate binding loop (Gly83, Ser84). Interactions with key residues in the substrate binding site are picked up by the isophthalic acid moiety (Arg230, Ser222, Thr195 and Thr196) (Fig.   7E). The recapitulation of key interactions between substrate and protein supports the conclusion that these ligands likely bind to and inhibit mycobacterial MurE, although the inherent flexibility of the Mur ligases is likely to complicate this picture. Experimental evidence is needed to confirm inhibition of mycobacterial Mur ligases by any of the inhibitors discussed here.

As can be seen from this survey of the literature, much progress towards the development of potent inhibitors of the Mur ligases has been made. However, the translation from effective enzyme inhibition to antibacterial action is a significant challenge. Membrane permeability and active efflux are likely to present the biggest hurdles and many inhibitors developed to date are very polar, particularly the phosphinate transition state analogues and amino acid derivatives, making them unlikely to easily traverse the membrane (Jarlier and Nikaido 1994). For other classes of inhibitors, the explanation is less clear.

It is also important to highlight that for those compounds with a demonstrated antibacterial activity there is often no evidence for the observed bactericidal effect being caused by inhibition of a Mur ligase (Silver 2011). The IC_50_/MIC data in conjunction with molecular modelling or X-ray crystallography, would significantly inform the design of next generation of inhibitors. The study by Barreteau *et al*. (Barreteau *et al*. 2012), which evaluated a selection of inhibitors designed to target MurD from *E. coli* against orthologues from different bacteria is a step in this direction.

Many of the inhibitors discussed contain what would typically be considered undesirable functional groups, for example rhodanine and thiazolidindione moieties are notorious pan assay interference compounds (PAINS) (Baell 2010; Baell and Holloway 2010). These compounds often prove to be not amenable to optimisation, preventing the progression of hit/lead molecules. The pitiful success of HTS as a source of novel antibacterial agents has been the subject of a comprehensive commentary in the literature (Tomašić and Mašič 2012). Hits derived from HTS campaigns are typically quite lipophilic, whereas antibiotics are typically larger and more polar than drugs designed for human targets (Payne *et al*. 2007). It is, therefore, questionable whether the libraries amassed by the pharmaceutical industry are optimal for antibacterial drug discovery.

Fragment-based drug discovery may offer a viable alternative (Hajduk and Greer 2007). This approach employs libraries of low molecular weight compounds or fragments, these could include commercial fragment libraries, repurposed kinase focussed libraries to target the ATP binding site and libraries of phosphate bioisosteres (Huang *et al*. 2009; Volkamer *et al*. 2015). The fragments are screened against the protein targets of interest using a range of biophysical assays which have a much lower false positive hit rate compared to the biochemical assays typically used in HTS screens (Schulz and Hubbard 2009). One of the major advantages of this approach, and which is particularly relevant to antimicrobial drug discovery, is that due to the nature of the hits identified, i.e. low molecular weight, low-affinity and high-ligand efficiency, one can exert greater control over the physiochemical properties of the inhibitor during the process of hit to lead development (Hajduk 2006). The process of fragment optimisation is typically design intensive and structure guided, however, given the current lack of structural information for the mycobacterial enzymes, *in situ* optimisation using kinetic target guided synthesis has great potential and would allow rapid exploration of the protein binding landscape and the inherent flexibility of the Mur ligases (Hajduk 2006).

It is clear from the progress to date that to obtain anti-tubercular agents that target the Mur ligases a number of hurdles need to be overcome. As a result, further exploration of the Mur ligases as drug targets and the mechanisms of the inhibitors developed to date are necessary if therapeutically useful molecules are to be developed.

## CONCLUSIONS AND FUTURE OUTLOOK

Due to the protective role of PG and its absence in humans, its biosynthetic pathway remains an excellent therapeutic target (Strancar *et al*. 2007; Zawadzke *et al*. 2008; Tomasic *et al*. 2010). Encouragingly, technologies for high throughput screening against these targets have advanced (Kristan *et al*. 2009; Turk *et al*. 2009; Guzman *et al*. 2013a). We would hope that these developments and the new insights into the molecular mechanisms of cell wall synthesis could lead to the development of targeted anti-mycobacterials.

## Supplementary Material

fuz016_Supplement_Table_1
