# Supplementary material for: Cell wall peptidoglycan in Mycobacterium tuberculosis: An Achilles’ heel for the TB-causing pathogen
Source: FEMS Microbiol Rev. 2019 Jun 10;43(5):548–75. doi: 10.1093/femsre/fuz016 (PMC6736417; doi:10.1093/femsre/fuz016)
Supplement: fuz016_Supplement_Table_1 [file fuz016_supplement_table_1.docx]

**Supplementary Table 1. Function, substrates and inhibitors of mycobacterial PG enzymes.**

GlcN, glucosamine; Glc*N*Ac, *N-*acetylglucosamine; Mur*N*Ac, *N*-acetylmuramic acid; NR, not reported; PEP, phosphoenolpyruvate; UDP, uridine diphosphate; UTP, uridine triphosphate.*Where *M. tuberculosis* specific inhibitors are not known, those targeting homologues in other bacterial species have been mentioned.

| **Enzyme (PDB)** | **Name** | **Function** | **Natural Substrate(s)** | **Inhibitors*** |
| --- | --- | --- | --- | --- |
| **GlmS** | Amidotransferase | Converts d-fructose-6-phosphate to d-Glc*N*-6-phosphate | d-fructose-6-phosphate, l-Glu | Glutamine analogues |
| **GlmM** | Phosphoglucosaminemutase | Converts Glc*N*-6-P to Glc*N*-1-P | Glc*N*-6-P | Phosphatase |
| **GlmU (3D98, 3D8V)** | Glucosamine-1-P acetyl transferase and *N*-acetylglucosamine-1-P uridyltransferase | Converts GlcN-1-P to UDP-Glc*N*Ac | Glc*N*-1-P, UTP | Phosphatase,  aminoquinazoline-based |
| **MurA** | UDP-*N*-acetylglucosamine enolpyruvyl transferase | Transfers PEP to UDP-*N*-acetylglucosamine | PEP, UDP-*N*-acetylglucosamine | Fosfomycin (ineffective for *M. tuberculosis*) |
| **MurB (5JZX)** | UDP-*N*-acetylenolpyruvylglucosamine reductase | Reduces enolpyruvyl-UDP-*N*-actylglucosamine to UDP-*N*-acetylmuramic acid | Enolpyruvyl-UDP-*N*-actylglucosamine, NADPH | 4-thiazolidinones, disulphate surrogates, 3,5-dioxopyrrolidin |
| **MurC** | Murein peptide ligase C | Ligates l-Ala to UDP-Mur*N*Ac | l-Ala, ATP | Phosphinate, l-Ala analogues |
| **MurD** | Murein peptide ligase D | Ligates d-Glu to UDP-Mur*N*Ac-l-Ala | d-Glu, ATP | Phosphinate, d-Glu analogues |
| **MurE (2XJA, 2WTZ)** | Murein peptide ligase E | Ligates *m*DAP to UDP-Mur*N*Ac-l-Ala-d-Glu | UDP-Mur*N*Ac-l-Ala-d-Glu, *m*DAP, ATP | Phosphinate, *m*DAP analogues |
| **MurF** | Murein peptide ligase F | Ligates d-Ala-d-Ala to UDP-Mur*N*Ac-l-Ala-d-Glu-*m*DAP | d-Ala-d-Ala, ATP | Aminoalkylphosphinate |
| **Ddl (3LWB)** | d-Ala:d-Ala ligase | ligates two d-Ala to d-Ala-d-Ala | d-Ala, ATP | d-cycloserine, d-Ala analogues |
| **Alr**  **(1XFC)** | Alanine racemase | Converts l-Ala into d-Ala | l-Ala | d-cycloserine, thiadiazolidinones |
| **MraY** | Phospho-*N*-acetyl muramoylpentapeptide transferase (translocase I) | Transfers phospho-*N*-acetylmuramic acid pentapeptide to decaprenyl phosphate, obtaining lipid I | *N*-acetyl muramoyl pentapeptide; decaprenyl phosphate | Nucleoside based inhibitors (eg. Capuramycins) |
| **MurG** | Glycosyl transferase | Transfer of UDP-GlcNAc to lipid intermediate I | LipidI, UDP-GlcNAc | Transition state mimics |
| **MurI (5HJ7, 5IJW)** | Glutamate racemase | Converts l-Glu to d-Glu | l-Glu | Glu analogues (β-Chloro-d-Ala) |
| **GatD/**  **MurT** | Glutamine amido transferase/Murein ligase | Amidates D-Glu in the peptide stem of Lipid II to D-Gln | LipidII, l-Glu, ATP | NR |
| **FtsW/**  **RodA/ MurJ** | Putative flippases | Enable transport of PG precursors to the periplasm | Lipid II | NR |
| **Class A PBP** | Bifunctional Penicillin binding proteins | Glycosyltransfer and transpeptidation (DD-crosslinks) | Lipid II, nascent PG | glycopeptides, β-lactams |
| **Class B PBP** | Monofunctional Penicillin binding proteins | Transpeptidation (DD-crosslinks) | Nascent PG | β-lactams |
| **Ldt (5E5L, 5D7H, 5DU7 6D4K)** | LD-transpeptidases | forms LD-crosslinks | Nascent PG | Carbapenem (Imipenem) |
| **DacB (4P0M)** | DD-carbox/endoypeptidase | Removes d-Ala (position 5 of stem peptide) and/or cleaves DD-cross-links | PG | Carbapenem (Meropenem) |
| **NagA** | *N*-acetylglucosamine-6-phosphate deacetylase | Converts GlcNAc-6-P to GlcN-6-P | GlcNAc-6-P | transition state inhibitor (eg. *N*-methylhydroxyphosphinyl-d -glucosamine-6-phosphate) |
| **NagZ** | *N*-acetylglucosaminidase | Cleaves the bond between GlcNAc and MurNAc | PG fragments | Β-glucosaminidase inhibitor,  O-(2-acetamido-2-deoxy-d-gluco  pyranosylidene)amino *N*-phenylcarbamate |
